# How the scientific community responded to the COVID-19 pandemic: A subject-level time-trend bibliometric analysis

**DOI:** 10.1371/journal.pone.0258064

**Published:** 2021-09-30

**Authors:** Mohammad-Reza Malekpour, Mohsen Abbasi-Kangevari, Sina Azadnajafabad, Seyyed-Hadi Ghamari, Negar Rezaei, Sahba Rezazadeh-Khadem, Nazila Rezaei, Arya Aminorroaya, Elham Abdolhamidi, Sahar Mohammadi Fateh, Rosa Haghshenas, Shahin Roshani, Naser Ahmadi, Kosar Jamshidi, Shohreh Naderimagham, Farshad Farzadfar

**Affiliations:** 1 Non-Communicable Diseases Research Center, Endocrinology and Metabolism Population Sciences Institute, Tehran University of Medical Sciences, Tehran, Iran; 2 Social Determinants of Health Research Center, Shahid Beheshti University of Medical Sciences, Tehran, Iran; 3 Endocrinology and Metabolism Research Center, Endocrinology and Metabolism Clinical Sciences Institute, Tehran University of Medical Sciences, Tehran, Iran; University of Central Florida, UNITED STATES

## Abstract

**Background:**

COVID-19 has triggered an avalanche of research publications, the various aspects of which need to be assessed. The objective of this study is to determine the scientific community’s response patterns to COVID-19 through a bibliometric analysis of the time-trends, global contribution, international collaboration, open-access provision, science domains of focus, and the behavior of journals.

**Methods:**

The bibliographic records on COVID-19 literature were retrieved from both PubMed and Scopus. The period for searching was set from November 1, 2019, to April 15, 2021. The bibliographic data were coupled with COVID-19 incidence to explore possible association, as well as World Bank indicators and classification of economies.

**Results:**

A total of 159132 records were included in the study. Following the escalation of incidences of COVID-19 in late 2020 and early 2021, the monthly publication count made a new peak in March 2021 at 20505. Overall, 125155 (78.6%) were national, 22548 (14.2%) were bi-national, and 11429 (7.2%) were multi-national. Low-income countries with 928 (66.8%) international publications had the highest percentage of international. The open-access provision decreased from 85.5% in February 2020 to 62.0% in April 2021. As many as 82841 (70.8%) publications were related to health sciences, followed by life sciences 27031 (23.1%), social sciences 20291 (17.3%), and physical sciences 15141 (12.9%). The top three medical subjects in publications were general internal medicine, public health, and infectious diseases with 28.9%, 18.3%, and 12.6% of medical publications, respectively.

**Conclusions:**

The association between the incidence and publication count indicated the scientific community’s interest in the ongoing situation and timely response to it. Only one-fifth of publications resulted from international collaboration, which might lead to redundancy without adding significant value. Our study underscores the necessity of policies for attraction of international collaboration and direction of vital funds toward domains of higher priority.

## Introduction

The COVID-19 pandemic continues to place an extraordinary burden on humankind. During the pandemic of an emerging infectious disease, it is essential yet challenging to acquire the maximum solid knowledge as fast as possible. Therefore, there have been a significant allocation of research funding and changes in the publication process to facilitate the knowledge spread, which have triggered an avalanche of publications on COVID-19 [1]. The accelerated speed of publications could ensure the prompt sharing of the new evidence; however, dissemination of a large volume of publications has also raised some concerns [2].

Although the pandemic has inversely affected various aspects of human lives, most publications on COVID-19 have reportedly focused on the field of medicine. In contrast, other domains of science have so far received less scientific attention [3]. The research priorities need to be in line with the world’s current needs as imposed by the pandemic [4]. Moreover, the scientific community should also not get side-tracked by the race in publishing articles. However, we should specifically pay attention to the audience among policymakers, who need to make rapid evidence-based decisions [5].

Containment of the pandemic calls for international collaboration to improve the understanding of the situation [6]. Nevertheless, there has been a reduction in the rate of international collaborations during the pandemic, which could have consequences for the quality of literature and the direction of future researches [7]. Looking at the pattern of publications on the previous emerging infections, Severe Acute Respiratory Syndrome (SARS) and Middle East Respiratory Syndrome (MERS), there was significant heterogeneity in international collaboration [8]. In addition, the time-trends in publishing research articles on SARS and MERS indicate that the scientific community has shown fluctuating interest in the subject, which was associated with the incidence dynamics of the outbreaks [1, 9], rather than the annual list of priority diseases published by World Health Organization (WHO) [10].

Therefore, there needs to be constant monitoring of the research publications regarding the time-trends, global contribution, international collaboration, and subjects of literature published during the pandemic. These insights into the characteristics of the current body of literature on COVID-19 can help policymakers develop strategies to respond appropriately to public health emergencies. The objective of this study is to identify the scientific community’s response patterns to the COVID-19 pandemic by providing a bibliometric analysis of the existing literature on COVID-19 as indexed by Scopus and PubMed from the early days of the pandemic until April 15, 2021.

## Materials and methods

The bibliographic data on COVID-19 literature was retrieved from PubMed and Scopus, two mainstream scholarly databases that contain publications from peer-reviewed journals. Although preprint literature provides the immediate distribution of results, we did not include preprint databases since the lack of quality control could increase the risk of invalid information dissemination, especially during the outbreaks [8].

Both databases were mined using their official Application Programming Interface (API) with queries shown in S1 Table. The acceptance dates for more than half of the records were unavailable. There were also many electronically published papers with printed publication dates far beyond their indexing date. Therefore, the indexing date in PubMed or Scopus was considered as the date parameter. The period for searching was set from November 1, 2019, to April 15, 2021. A language restriction for English was placed, and document types other than original research, review, note, letter, and comment were excluded. To have a better estimation of the indexing time of articles in PubMed, the time intervals between acceptance and indexing date for literature with available acceptance dates were calculated. The global incidence of COVID-19 was retrieved from the GitHub repository provided by Johns Hopkins University [11]. Besides, the Research and Development (R&D) expenditure of countries, as well as the number of annual scientific publications, were retrieved from World Bank [12–14].

After unifying journal and affiliation names, we merged PubMed and Scopus datasets and removed duplicates based on the digital object identifier (DOI). Then, seven features were chosen for analysis, including indexing date, document type, journal, affiliation, citation count, subject area, and open-access status. As PubMed’s API did not provide the country included in each affiliation, we used the Geotext Python library to extract countries from affiliations [15]. Afterward, each paper was assigned to all of its affiliations and corresponding countries. Moreover, the countries were categorized as different regions and income groups based on the World Bank classification of economies [16].

Since PubMed’s API did not provide any metadata about papers’ topics and open-access status, we used the Scopus dataset for the related analysis. As defined by Scopus, the four general domains of science included health sciences, life sciences, social sciences, and physical sciences [17]. The medical subjects in the health sciences were categorized as presented in S2 Table.

The monthly incidence of COVID-19 was coupled with publication count and plotted against date to explore their time-trends and possible association. To investigate international collaborations, papers were categorized as national, bi-national, and multi-national based on the number of countries involved. We also performed network analysis to compute the Degree and the Normalized Degree Centrality of countries using NetworkX [18], a Python library for studying graphs and networks. The Degree measures the total number of connections between countries. The Normalized Degree Centrality for a particular country indicates the number of other distinct countries it is connected to, divided by n-1, where n is the number of total countries. Moreover, to include publication count effect on the Degree, we computed Degree to publications ratio by dividing each country’s Degree value by its publication count.

Python programming language, version 3.6 [19], was utilized for data mining, preprocessing, and analysis. Data visualizations were performed using Tableau Desktop, version 2020.1 [20], an interactive data visualization software.

## Results

### Overview

Our data extraction retrieved 111074 bibliometric records from PubMed and 117012 records from Scopus. After omitting duplicates, a total of 159132 records were included. Totally, 107584 (67.6%) were original research, 18380 (11.5%) letter, 18304 (11.5%) review, 7596 (4.8%) comment, and 7268 (4.6%) editorial. Of 70513 PubMed records for which acceptance dates were available, the median (IQR) time interval between acceptance and indexing date was 21 (11–42) days.

### Publication time-trends

**Fig 1:**
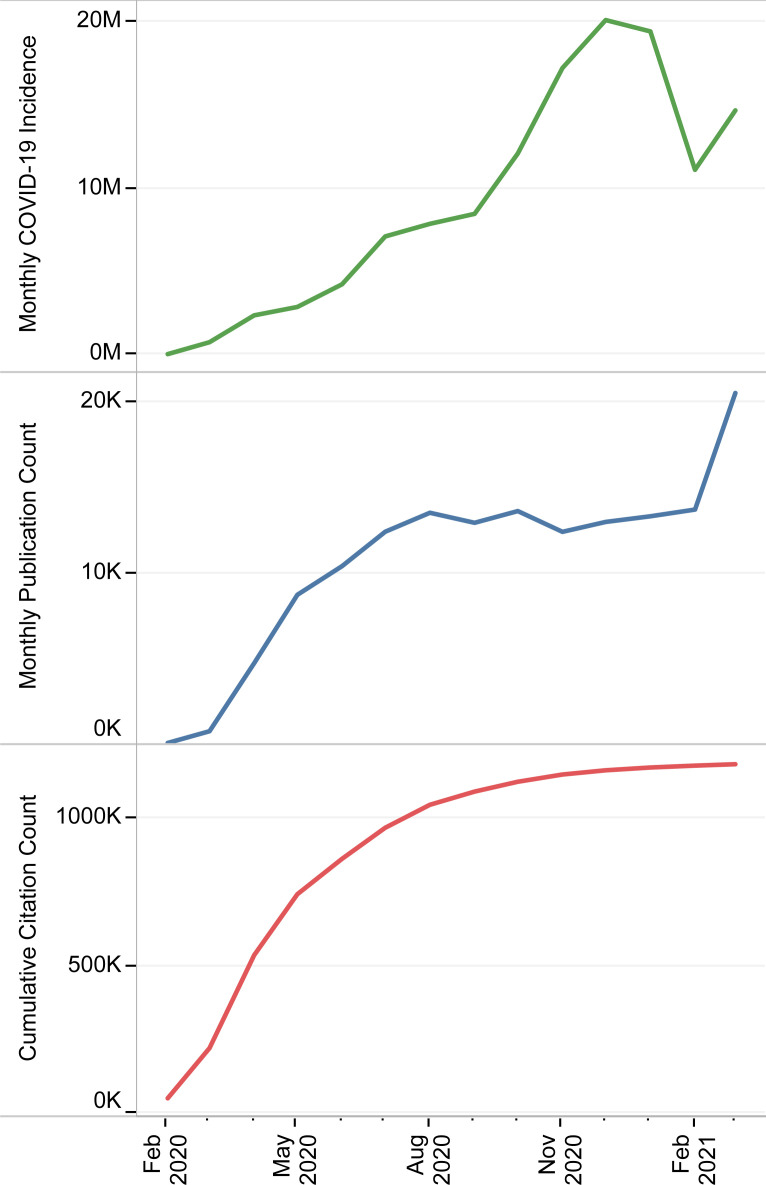
Monthly trends of global COVID-19 incidence, publications, and cumulative count of citations.

**Fig 2:**
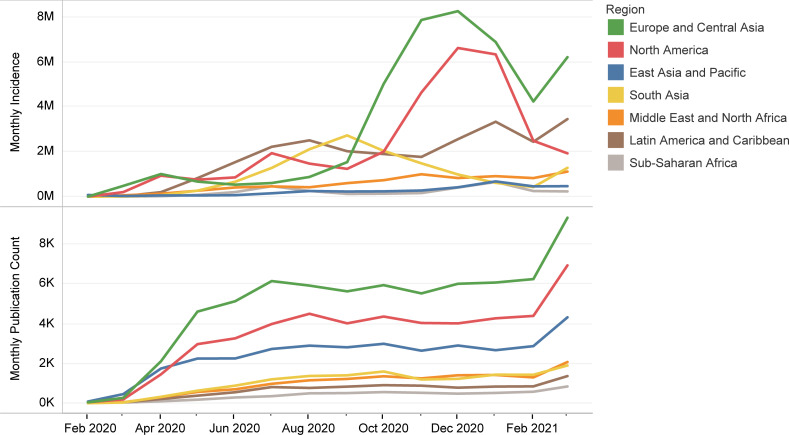
Monthly trends of global COVID-19 incidence and publications for each World Bank region.

Coinciding with the sharp growth of monthly confirmed COVID-19 cases, the monthly publication count increased dramatically in the second trimester of 2020, reaching a relative plateau for the rest of 2020 (Fig 1). However, following the escalation of global incidences of COVID-19 in late 2020 and early 2021, the monthly publication count rose again in March 2021, which resulted in a new peak at 20505. Despite an almost linear increase in the number of total publications, the cumulative count of citations formed a stationary state after a period of sharp growth. Except for the pandemic’s early days when the East Asia and Pacific region was in the lead, most articles originated from the Europe and Central Asia region, followed by North America and the East Asia and Pacific region (Fig 2). Nevertheless, the monthly trends of publication count were almost the same and independent of incidence rates in different regions.

### Global contribution

**Fig 3:**
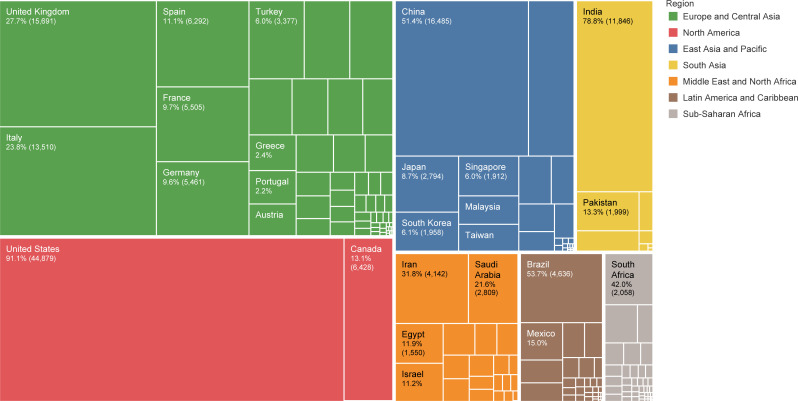
Share of countries in publications within their corresponding World Bank regions.

With contribution to 28.2% of all papers, the United States (US) had the largest share of publications, followed by China with 10.4% and the United Kingdom (UK) with 9.9%. Moreover, China, the US, and the UK, with 34.1%, 31.3%, and 12.8% of total citations, respectively, were the top three countries in this regard as well (S3 Table). The leading 20 research institutes in publications were based in eight countries, including the US, Canada, China, the UK, Italy, Iran, France, and Pakistan (S4 Table). The UK (27.7%), the US (91.1%), China (51.4%), India (78.8%), Iran (31.8%), Brazil (53.7%), and South Africa (42.0%) had the highest share of publications in their corresponding regions (Fig 3 and S5 Table). The leading countries in each income group were the US (high-income), China (upper-middle-income), India (lower-middle-income), and Ethiopia (low-income) with contributing to 42.3%, 42.7%, 58.4%, and 33.9% of publications in their region, respectively (S6 Table and S1 Fig). Countries with at least 1000 publications related to COVID-19 were ranked on the ratio of COVID-19 publications to R&D expenditure, where Pakistan, Nigeria, and Iran were the top three countries (Table 1).

**Table 1:** The top ten countries in COVID-19 publications to R&D expenditure ratio among settings with at least 1000 COVID-19 publications.

	Publications	R&D Expenditure	Publications to R&D expenditure Ratio
Pakistan	1,999	$0.657B	3,045
Nigeria	1,034	$0.593B	1,745
Iran	4,142	$3.769B	1,099
Egypt	1,550	$2.194B	706
South Africa	2,058	$2.924B	704
India	11,846	$18.647B	635
Greece	1,356	$2.471B	549
Indonesia	1,246	$2.533B	492
Italy	13,510	$28.032B	482
Turkey	3,377	$7.318B	461

Bangladesh, with 33.0%, had the highest ratio of COVID-19 publications to the annual scientific publications among countries with at least 1000 COVID-19 publications, followed by Saudi Arabia (25.8%), and Ireland (19.0%); In contrast, Japan (2.8%), South Korea (2.9%), and China (3.1%) had the lowest percentages (S7 Table).

### International collaboration

**Fig 4:**
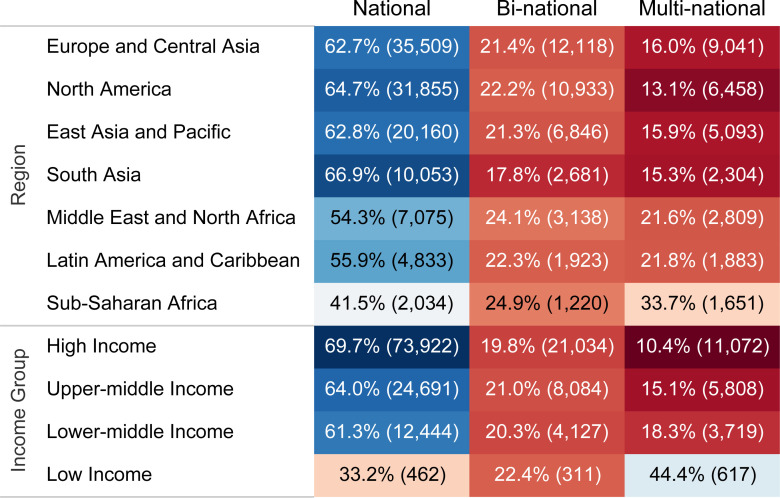
Distribution of the national, bi-national, and multi-national collaboration in World Bank regions and income groups.

Of all publications, 125155 (78.6%) were national, 22548 (14.2%) were bi-national, and 11429 (7.2%) were multi-national. However, the international collaboration varied considerably among regions and income groups. Sub-Saharan Africa, with 2871 (58.5%) international papers, had the highest percentage of international collaboration among regions. In addition, low-income countries with 928 (66.8%) international publications had a significantly higher percentage of international collaboration than other income groups, ranging from 30.2% to 38.6% (Fig 4). Among the countries with at least 1000 publications related to COVID-19, Switzerland with 2118 (74.7%) international publications, followed by Sweden 1184 (74.4%) and Denmark 785 (71.0%) were the top three countries in the percentage of international collaboration (S2 Fig).

The most collaborating countries were the US, the UK, and Italy, with Degree values of 31668, 21517, and 15529, respectively. Taking the total publication count into account, the US with Degree to publications ratio of 1.42 was the most collaborating among countries with at least 1000 publications on COVID-19, followed by China (1.40), and India (1.38). The UK-US, the China-US, and the Italy-US were the most collaborative pairs with 3112, 2611, and 2284 joint publications related to COVID-19, respectively. In terms of the Normalized Degree Centrality, the US had collaborated with 92.0% of countries, followed by the UK (82.6%), Italy (76.1%) (Table 2 and S8 Table).

**Table 2:** The Degree, publications, Degree to publications ratio, and the Normalized Degree Centrality of the leading ten countries in COVID-19 publications.

	Degree	Publications	Degree to Publications Ratio	Normalized Degree Centrality
United States	31,668	44,879	1.42	92.0%
United Kingdom	21,517	15,691	0.73	82.6%
Italy	15,529	13,510	0.87	76.1%
China	11,803	16,485	1.40	70.9%
Germany	10,967	5,461	0.50	65.3%
Canada	10,009	6,428	0.64	71.8%
Australia	9,969	5,655	0.57	70.9%
Spain	9,819	6,292	0.64	71.4%
France	9,465	5,505	0.58	74.6%
India	8,571	11,846	1.38	74.6%

### Open-access provision

Among 117012 publications indexed in Scopus, 85354 (72.9%) were open-access. The percentage of publications with open-access decreased from 85.5% in February 2020 to 62.0% in April 2021 (S3 Fig). The order of the income groups in the percentage of open-access provision was low-income (83.0%), upper-middle-income (75.1%), high-income (74.3%), and lower-middle-income (69.7%). Besides, 78.7% of multi-national articles, 76.0% of bi-national, and 71.8% of national publications were open-access (S4 Fig). Among countries with at least 1000 COVID-19 publications, Sweden (86.2%), Netherlands (82.7%), and Brazil (82.0%%) had the highest percentage of open-access provision (S9 Table).

### Science domains and medical subjects

As many as 82841 (70.8%) publications were related to health sciences, followed by life sciences 27031 (23.1%), social sciences 20291 (17.3%), and physical sciences 15141 (12.9%). It is worth mentioning that 29804 (25.5%) COVID-19 articles were related to more than one domain. Regarding total citations for each domain, 860440 (78.4%) related to health sciences, 280745 (25.6%) to life sciences, 85828 (7.8%) to physical sciences, and 71099 (6.5%) to social sciences.

**Fig 5:**
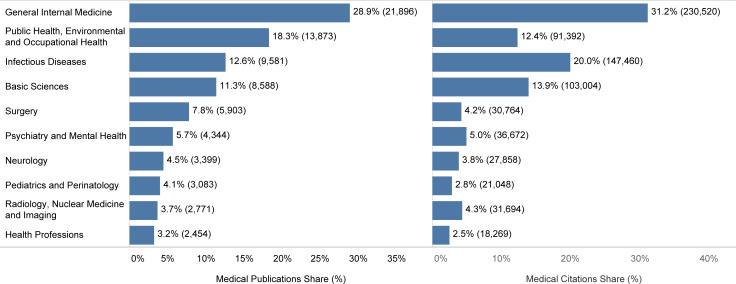
Share of the top ten medical subjects in the number of medical publications and citations.

**Fig 6:**
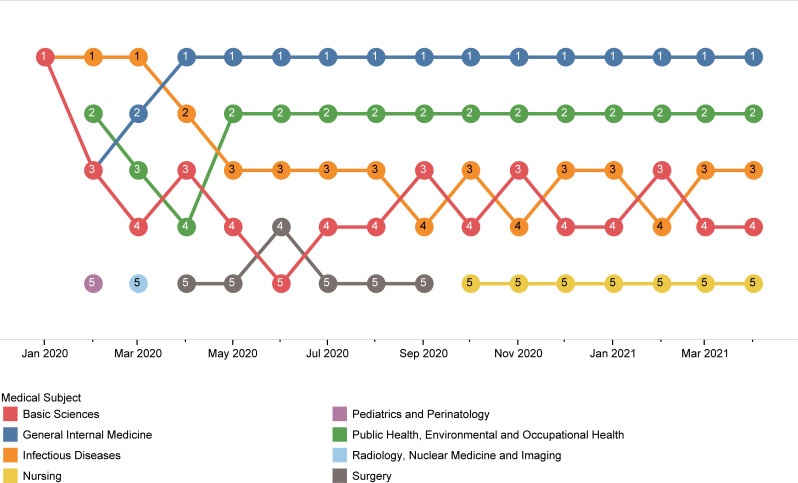
Monthly ranking of the top five medical subjects in publications.

**Fig 7:**
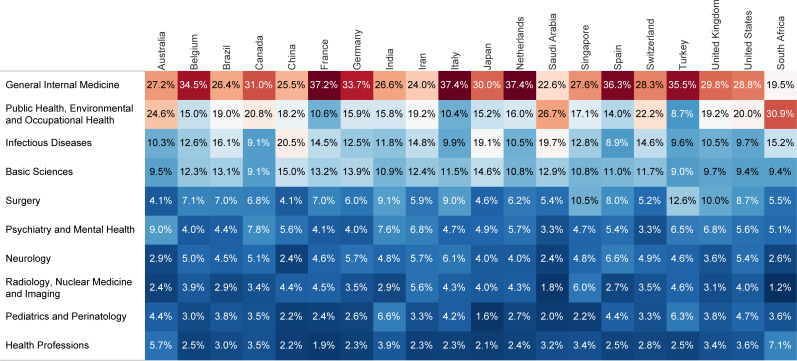
Distribution of the publications among the top ten medical subjects for the 20 leading countries in publication count.

Among the medical subjects, general internal medicine with 21896 (28.9%) publications and 230520 (31.2%) citations, was the leading one, both in the number of publications and citations. Although public health with 13873 (18.3%) ranked second in publication count, it did not rank better than fourth with 91392 (12.4%) in citation count (Fig 5). The monthly ranking of the top five medical subjects in publication count demonstrated the changing pattern of interest in the early days of the pandemic (Fig 6). Nevertheless, general internal medicine and public health had been given the most attention throughout the period of this study. Moreover, there were almost the same distributions of different medical subjects among the leading 20 countries in publication count, with general internal medicine getting the most attention, followed by public health and infectious diseases (Fig 7). South Africa, Saudi Arabia, and Australia put the most effort into public health, with 30.9%, 26.7%, and 24.6% of their COVID-19 publications in medical subjects related to public health, respectively.

### Journals contribution

Only one journal, Journal of Medical Virology was listed in the top ten journals both in publications and citations (S10 and S11 Tables). Among journals with more than 100 publications on COVID-19, New England Journal of Medicine (NEJM) with 157.3 had the highest citations to articles ratio, followed by The Lancet (136.7), and Cell (103.3) (Table 3).

**Table 3:** The top ten journals in citations to articles ratio among journals with more than 100 publications on COVID-19.

	Citations	Publications	Citations to Articles Ratio
New England Journal Of Medicine	67,015	426	157.3
The Lancet	67,006	490	136.7
Cell	14,464	140	103.3
Radiology	12,509	123	101.7
JAMA	43,635	502	86.9
Science	18,210	211	86.3
Clinical Infectious Diseases	12,211	172	71.0
The Lancet Respiratory Medicine	12,458	177	70.4
The Lancet Infectious Diseases	15,488	238	65.1
International Journal Of Antimicrobial Agents	7,993	128	62.4

As many as 66.8% of publications in the BMJ had at least one author affiliated to the UK. Similarly, the US had contributed to 66.1% and 60.1% of COVID-19 articles published in JAMA and Cureus, respectively (S5 Fig). The most prolific journals in each general domain of science are reported in S12 Table.

## Discussion

### Overview

In this study, we extracted 159132 publications to investigate the response patterns of the scientific community to the COVID-19 pandemic. There was a relative association between the monthly COVID-19 incidence and publication count, which indicated the dependence of the scientific community’s interest on the ongoing situation. The monthly publication count increased dramatically about two months after WHO had proclaimed COVID-19 to be a Public Health Emergency of International Concern (PHEIC) [21]. In addition to the peer-reviewing, this delay could partly be due to the indexing process, which was shown for PubMed to be at least 3 weeks for more than half of articles. Considering these two hindrances, we can infer that the scientific community responded appropriately quickly, which was consistent with the findings of studies related to other PHEICs [3, 8, 22].

### International collaboration

The international collaboration may offer increased scientific capacity and output in confronting PHEICs. While research collaboration benefits low-income countries with resources and competencies, it can also boost high-income countries’ performance in publications via providing timely access to emerging data; However, with only about one-fifth of COVID-19 publications resulting from international collaboration, there was not any improvement in this figure during the pandemic compared with before [23]. This could be due to barriers to sharing ideas and data imposed by the lockdown or low available resources for international collaboration, which might increase research redundancy without added value. Low-income countries had a considerably higher international collaboration, which emphasizes the need for resources in these countries. Notably, international collaboration percentage in COVID-19 publications varied widely, even among the most prolific countries, from about three quarters in Switzerland and Sweden to one quarter in India and Turkey. It seems that countries’ tendency toward international collaboration during the COVID-19 era was almost the same as what it was under normal circumstances; for instance, Switzerland and Turkey had 71% and 24% international collaboration in overall publications in 2018, respectively [23].

The US with the highest Degree to publications ratio and at least one joint publication with 92.0% of countries, played a key role in international collaboration. On the other hand, as the pandemic’s primary epicenter, a significant proportion of the early data about COVID-19 originated from China, a collaboration opportunity for Chinese and international researchers. China and the US have played a significant role in the global network of COVID-19 research, which resulted in increased bilateral research articles during the pandemic by about 36% [7].

### Open-access provision

Despite the necessity of providing open-access to scientific reports, our results showed a decreasing trend in the percentage of open-access publications from 85.5% in February 2020 to 62.0% in April 2021. Scientific communication and open data offer key building blocks to create a robust knowledge base. Therefore, it is imperative to guarantee the open-access and inclusiveness of publications and emerging data, at least during the pandemics. Unaffordability of journal fees and subscriptions, particularly towards low and lower-middle-income countries, would hinder many countries’ participation in global science and limit their contribution to international research [24]. Fortunately, the waivers that most journals have for low-income countries have led to the highest percentage of open-access provision in COVID-19 literature by low-income countries. Nonetheless, lower-middle-income countries did not appear to have benefited from these waivers.

### Science domains and medical subjects

Since the beginning of the pandemic, most publications belonged to the health sciences, which also formed the most-cited literature. Nevertheless, the other domains, including life, social, and physical sciences, have increasingly drawn researchers’ attention. In the early days, it was expected that the spotlight be on the pathogenesis of the SARS-CoV-2, prevention, or treatment [25]. However, the pandemic could have lasting negative impacts on the various aspects of human lives, though it was primarily considered a public health crisis [26]. Therefore, a more collaborative interdisciplinary effort is needed to address the uncertainties regarding the aspects that have received improper attention. Over time, the scientific community showed varying interests in medical subjects. General internal medicine had the highest number of publications and citations, followed by public health and infectious diseases. The pandemic has indeed highlighted the essential role of public health in providing practical solutions and mitigation strategies to maintain community health based on the existing knowledge [27]. This calls for the active participation of countries’ public health experts, resulting in the publication of articles in the field as by-products. Nevertheless, there was significant heterogeneity in the proportion of literature related to public health published by countries, which varied from 30.9% in South Africa to 8.7% in Turkey. Therefore, it could be suggested that countries’ research priorities get reviewed, and countries reconsider their attitudes towards public health.

### Journals contribution

The top-cited articles were published in high-profile journals, including NEJM, The Lancet, and Cell. The citations to articles ratio of NEJM was the highest, followed by The Lancet, consistent with previous reports [28]. This could be since authors would likely find these distinguished journals more reliable for citation [29]. In addition to reliability, more concentration on concerning topics of COVID-19 and higher attachment to providing open-access were likely the leading causes of witnessed higher citation rates of articles published in these journals [30].

There was an unexpected increase in the number of articles published in some journals, which were not necessarily high-profile. This could particularly become threatening, as some journals might see the pandemic as a golden opportunity to gain reputation or financial benefit. Besides, analyzing the country of origin among the journals with the greatest number of publications revealed a possible selection bias toward some countries. This could confine international contribution to scientific globalization and may lead to missing opportunities for coping with the pandemic on a global scale [31].

### Policymaking

This pandemic’s immense and abrupt burden has resulted in unorganized research funding in many areas of science, especially medicine, and almost in all countries facing the disease. This chaotic condition has resulted in the mass production of so-called "waste" in research due to low-quality research questions, designs, and reports of results [32]. These publications not only waste research resources but can also be misleading and potentially harmful, such as two retracted papers by The Lancet and NEJM [33, 34]. Time insufficiency and defective research basis in the COVID-19 era even amplify flawed research, mainly because of the rush to research in many science areas, especially medicine that tries to sedate the communities [35]. To successfully reduce research biases and waste of funding and financial support of researchers, multidisciplinary research priorities need to be deployed to guide the scientific community [4]. A critical step in such planning is developing and implementing an evidence-based framework for research questions. Such a practical framework could easily show gaps in knowledge and lead researchers and funding agents to the appropriate direction [36]. Besides, vulnerable areas and populations like low and lower-middle-income countries need a distinct investigation and financial support of research. In most cases, they lack the proper research resources and adversely bear the disease’s greatest burden due to defects in the medical systems and reserves [37]. Therefore, we advise the scientists and the policymakers of all health and non-health related authorities to clarify the priorities of the COVID-19 research and make the vital funds and resources available for the destitute fields of studies and populations to finally make their endeavors merited.

### Strengths and limitations

In our study, 159132 publications on COVID-19 were retrieved from both PubMed and Scopus, two primary scholarly databases which were considered relatively comprehensive and representative. However, there still might be publications that were not in our scope. Language bias might also occur as a result of excluding non-English publications. Another noteworthy limitation that needs to be acknowledged to apprehend the findings is the unavailability of the subject area metadata and open-access status in PubMed’s API. Nevertheless, giving an illustration of the current situation could help scientists to tackle this pandemic in different aspects of science, the benefit of which could outweigh this limitation.

## Conclusions

The association between the COVID-19 incidence and publication count indicated the scientific community’s interest in the ongoing situation and timely response to it. Despite the necessity of multilateral efforts in the containment of pandemics, only about one-fifth of COVID-19 publications resulted from international collaboration. Besides, although scientific communication offers building blocks of knowledge-base, the trend of open-access provision was decreasing. In spite of the fact that the pandemic was primarily considered a public health crisis, there was significant heterogeneity in the proportion of literature related to public health among countries. Therefore, it could be suggested that research priorities for COVID-19 be reviewed, and essential policies be made to attract international collaboration and make vital funds available for domains of sciences of higher priority.

## Supporting information

S1 Table The data retrieval queries used in PubMed and Scopus APIs. (PDF)

S2 Table Groups of medical subjects in the health sciences domain. (PDF)

S3 Table Publication count, publication share, citation count, and citation share of countries with at least ten COVID-19 publications. (PDF)

S4 Table The top 20 research institute in publications along with their country and state/province. (PDF)

S5 Table Publication count, publication share, citation count, and citation share of countries within their corresponding World Bank regions. (PDF)

S6 Table Publication count, publication share, citation count, and citation share of countries within their corresponding World Bank income groups. (PDF)

S7 Table The COVID-19 publications to annual publications ratio of countries with at least 1000 COVID-19 publications. (PDF)

S8 Table The Degree, publications, Degree to publications ratio, and the Normalized Degree Centrality of countries with at least 1000 COVID-19 publications. (PDF)

S9 Table Percent of open-access provision among countries with at least 1000 publications on COVID-19. (PDF)

S10 Table The top 30 journals in publication count. (PDF)

S11 Table The top 30 journals in citation count. (PDF)

S12 Table Publication count, publication share, citation count, and citation share of journals with at least 0.5% share in publications of each general domain of science. (PDF)

S1 Fig Share of countries in publications within their corresponding income groups. (PDF)

S2 Fig Distribution of the national, bi-national, and multi-national collaboration of countries with at least 1000 publications related to COVID-19. (PDF)

S3 Fig Monthly trend of open-access provision. (PDF)

S4 Fig Percent of open-access publications in each region, income group, and international collaboration type. (PDF)

S5 Fig Country distribution of publications in the leading 30 journals and 20 countries in COVID-19 publications. (PDF)
