# Supplementary material for: How the scientific community responded to the COVID-19 pandemic: A subject-level time-trend bibliometric analysis
Source: PLoS One. 2021 Sep 30;16(9):e0258064. doi: 10.1371/journal.pone.0258064 (PMC8483337; doi:10.1371/journal.pone.0258064)
Supplement: S1 Table — (PDF) [file pone.0258064.s001.pdf]

Supplementary Table 1 The data retrieval queries used in PubMed and Scopus APIs

Database	Query
PubMed	covid19[TIAB] OR covid[TIAB] OR COVID-19[TIAB] OR 2019-ncov[TIAB] OR "2019 Novel Coronavirus"[TIAB] OR "Coronavirus Disease 2019"[TIAB] OR "SARS-CoV-2"[TIAB] OR covid-2019[TIAB] OR "2019 ncov"[TIAB] OR 2019ncov[TIAB] OR "severe acute respiratory syndrome coronavirus 2"[TIAB]
Scopus	TITLE-ABS(covid19 OR covid OR COVID-19 OR 2019-ncov OR "2019 Novel Coronavirus" OR "Coronavirus Disease 2019" OR "SARS-CoV-2" OR covid-2019 OR "2019 ncov" OR 2019ncov OR "severe acute respiratory syndrome coronavirus 2")
