# Supplementary material for: How the scientific community responded to the COVID-19 pandemic: A subject-level time-trend bibliometric analysis
Source: PLoS One. 2021 Sep 30;16(9):e0258064. doi: 10.1371/journal.pone.0258064 (PMC8483337; doi:10.1371/journal.pone.0258064)
Supplement: S2 Table — (PDF) [file pone.0258064.s002.pdf]

Supplementary Table 2 Groups of medial sub-topics in the health sciences domain

**Groups**	**Medical Subjects**
Anesthesiology and Pain Medicine	Anesthesiology and Pain Medicine
Basic Sciences	Anatomy<br>Biochemistry<br>Embryology<br>Genetics<br>Histology<br>Microbiology<br>Pharmacology<br>Physiology
Dermatology	Dermatology
Emergency Medicine	Emergency Medicine
Family Medicine	Family Practice
General Internal Medicine	Cardiology and Cardiovascular Medicine<br>Complementary and Alternative Medicine<br>Critical Care and Intensive Care Medicine<br>Endocrinology, Diabetes and Metabolism<br>Gastroenterology<br>Geriatrics and Gerontology<br>Hematology<br>Hepatology<br>Immunology and Allergy<br>Internal Medicine<br>Nephrology<br>Oncology<br>Pulmonary and Respiratory Medicine<br>Rheumatology
Infectious Diseases	Infectious Diseases
Neurology	Neurology
Obstetrics and Gynecology	Obstetrics and Gynecology<br>Reproductive Medicine
Ophthalmology	Ophthalmology
Orthopedics and Sports Medicine	Orthopedics and Sports Medicine<br>Rehabilitation
Otorhinolaryngology	Otorhinolaryngology
Pathology and Forensic Medicine	Pathology and Forensic Medicine
Pediatrics, Perinatology and Child Health	Pediatrics, Perinatology and Child Health
Psychiatry and Mental Health	Psychiatry and Mental Health
Public Health, Environmental and Occupational Health	Epidemiology<br>Health Informatics<br>Health Policy<br>Public Health, Environmental and Occupational Health
Radiology, Nuclear Medicine and Imaging	Radiology, Nuclear Medicine and Imaging
Surgery	Surgery<br>Transplantation<br>Urology
