# Supplementary material for: How the scientific community responded to the COVID-19 pandemic: A subject-level time-trend bibliometric analysis
Source: PLoS One. 2021 Sep 30;16(9):e0258064. doi: 10.1371/journal.pone.0258064 (PMC8483337; doi:10.1371/journal.pone.0258064)
Supplement: S3 Table — (PDF) [file pone.0258064.s003.pdf]

Supplementary Table 3

	Publication Count	Publication Share	Citation Count	Citation Share
United States	44,879	28.20%	376,897	31.26%
China	16,485	10.36%	411,151	34.10%
United Kingdom	15,691	9.86%	154,236	12.79%
Italy	13,510	8.49%	126,656	10.50%
India	11,846	7.44%	45,356	3.76%
Canada	6,428	4.04%	54,622	4.53%
Spain	6,292	3.95%	43,511	3.61%
Australia	5,655	3.55%	53,556	4.44%
France	5,505	3.46%	63,557	5.27%
Germany	5,461	3.43%	69,720	5.78%
Brazil	4,636	2.91%	22,635	1.88%
Iran	4,142	2.60%	20,598	1.71%
Turkey	3,377	2.12%	13,287	1.10%
Switzerland	2,834	1.78%	36,460	3.02%
Saudi Arabia	2,809	1.77%	15,421	1.28%
Japan	2,794	1.76%	22,549	1.87%
Netherlands	2,654	1.67%	38,399	3.18%
South Africa	2,058	1.29%	9,280	0.77%
Pakistan	1,999	1.26%	9,391	0.78%
South Korea	1,958	1.23%	18,069	1.50%
Belgium	1,924	1.21%	18,727	1.55%
Singapore	1,912	1.20%	28,295	2.35%
Sweden	1,591	1.00%	16,833	1.40%
Poland	1,560	0.98%	8,374	0.69%
Egypt	1,550	0.97%	6,585	0.55%
Israel	1,461	0.92%	9,939	0.82%
Malaysia	1,389	0.87%	4,867	0.40%
Ireland	1,365	0.86%	9,040	0.75%
Greece	1,356	0.85%	14,984	1.24%
Mexico	1,298	0.82%	7,287	0.60%
Taiwan	1,276	0.80%	11,111	0.92%
Portugal	1,257	0.79%	6,286	0.52%
Indonesia	1,246	0.78%	5,151	0.43%
Austria	1,185	0.74%	16,089	1.33%
Denmark	1,105	0.69%	14,159	1.17%
Nigeria	1,034	0.65%	3,062	0.25%
Bangladesh	1,034	0.65%	5,646	0.47%
Russian Federation	937	0.59%	12,984	1.08%
Norway	849	0.53%	7,709	0.64%
New Zealand	841	0.53%	6,897	0.57%
United Arab Emirates	819	0.51%	4,219	0.35%
Thailand	792	0.50%	5,276	0.44%
Colombia	780	0.49%	5,478	0.45%

Supplementary Table 3

	Publication Count	Publication Share	Citation Count	Citation Share
Romania	635	0.40%	2,965	0.25%
Argentina	631	0.40%	3,821	0.32%
Chile	627	0.39%	2,776	0.23%
Finland	601	0.38%	4,779	0.40%
Jordan	583	0.37%	2,331	0.19%
Qatar	553	0.35%	2,350	0.19%
Vietnam	549	0.34%	7,019	0.58%
Philippines	536	0.34%	1,688	0.14%
Peru	509	0.32%	3,181	0.26%
Morocco	496	0.31%	1,544	0.13%
Ethiopia	471	0.30%	1,170	0.10%
Lebanon	463	0.29%	2,467	0.20%
Czech Republic	447	0.28%	2,487	0.21%
Nepal	432	0.27%	2,972	0.25%
Iraq	412	0.26%	1,530	0.13%
Croatia	358	0.22%	2,197	0.18%
Kenya	347	0.22%	1,033	0.09%
Ghana	320	0.20%	804	0.07%
Hungary	319	0.20%	2,648	0.22%
Serbia	290	0.18%	801	0.07%
Venezuela	282	0.18%	2,004	0.17%
Slovenia	273	0.17%	1,839	0.15%
Oman	273	0.17%	2,634	0.22%
Ecuador	270	0.17%	1,679	0.14%
Cyprus	239	0.15%	1,211	0.10%
Tunisia	232	0.15%	1,156	0.10%
Georgia	228	0.14%	661	0.05%
Kuwait	226	0.14%	1,487	0.12%
Ukraine	221	0.14%	797	0.07%
Uganda	218	0.14%	776	0.06%
Slovakia	161	0.10%	469	0.04%
Cameroon	160	0.10%	714	0.06%
Sri Lanka	146	0.09%	796	0.07%
Algeria	141	0.09%	383	0.03%
Sudan	139	0.09%	619	0.05%
Lithuania	132	0.08%	945	0.08%
Uruguay	131	0.08%	424	0.04%
Bulgaria	129	0.08%	601	0.05%
Kazakhstan	119	0.07%	400	0.03%
Tanzania	118	0.07%	539	0.04%
Malta	113	0.07%	381	0.03%
Luxembourg	112	0.07%	737	0.06%
Bahrain	110	0.07%	252	0.02%

Supplementary Table 3

	Publication Count	Publication Share	Citation Count	Citation Share
Zimbabwe	103	0.06%	418	0.03%
Cuba	101	0.06%	148	0.01%
Palestine	99	0.06%	207	0.02%
Estonia	94	0.06%	1,151	0.10%
Senegal	91	0.06%	923	0.08%
Democratic Republic Of The Congo	87	0.05%	300	0.02%
Yemen	86	0.05%	295	0.02%
Bosnia And Herzegovina	86	0.05%	143	0.01%
Zambia	79	0.05%	270	0.02%
Malawi	75	0.05%	265	0.02%
Libya	73	0.05%	153	0.01%
Jamaica	73	0.05%	185	0.02%
Bolivia	68	0.04%	1,220	0.10%
Mozambique	65	0.04%	926	0.08%
Costa Rica	64	0.04%	232	0.02%
Panama	60	0.04%	1,063	0.09%
Brunei Darussalam	56	0.04%	482	0.04%
Rwanda	55	0.03%	159	0.01%
North Macedonia	53	0.03%	539	0.04%
Afghanistan	53	0.03%	107	0.01%
Albania	52	0.03%	236	0.02%
Nicaragua	48	0.03%	69	0.01%
Latvia	44	0.03%	95	0.01%
Honduras	44	0.03%	1,353	0.11%
Paraguay	43	0.03%	716	0.06%
Puerto Rico	42	0.03%	368	0.03%
Syria	40	0.03%	68	0.01%
Mali	40	0.03%	429	0.04%
Congo	39	0.02%	1,279	0.11%
Botswana	39	0.02%	119	0.01%
Iceland	38	0.02%	555	0.05%
Fiji	36	0.02%	93	0.01%
Burkina Faso	36	0.02%	49	0.00%
Azerbaijan	36	0.02%	195	0.02%
Sierra Leone	35	0.02%	59	0.00%
Guatemala	35	0.02%	157	0.01%
Mauritius	33	0.02%	384	0.03%
Benin	33	0.02%	93	0.01%
Gabon	31	0.02%	115	0.01%
Dominican Republic	31	0.02%	68	0.01%
Belarus	31	0.02%	275	0.02%
Uzbekistan	30	0.02%	31	0.00%
Trinidad And Tobago	28	0.02%	69	0.01%

Supplementary Table 3

	Publication Count	Publication Share	Citation Count	Citation Share
Myanmar	27	0.02%	49	0.00%
Guinea	27	0.02%	117	0.01%
Cambodia	27	0.02%	323	0.03%
Armenia	27	0.02%	162	0.01%
Madagascar	25	0.02%	58	0.00%
Gambia	24	0.02%	116	0.01%
Barbados	24	0.02%	29	0.00%
Kyrgyzstan	22	0.01%	34	0.00%
Montenegro	19	0.01%	23	0.00%
Grenada	19	0.01%	54	0.00%
Cote D'Ivoire	18	0.01%	386	0.03%
Namibia	17	0.01%	48	0.00%
El Salvador	17	0.01%	37	0.00%
Somalia	15	0.01%	61	0.01%
Liechtenstein	15	0.01%	39	0.00%
Bhutan	14	0.01%	22	0.00%
Togo	13	0.01%	26	0.00%
Swaziland	13	0.01%	41	0.00%
Papua New Guinea	13	0.01%	59	0.00%
Niger	13	0.01%	4	0.00%
French Guiana	13	0.01%	101	0.01%
Maldives	12	0.01%	161	0.01%
Gibraltar	12	0.01%	9	0.00%
Aruba	12	0.01%	114	0.01%
Saint Kitts And Nevis	11	0.01%	4	0.00%
Kosovo	11	0.01%	11	0.00%
Haiti	11	0.01%	19	0.00%
Mongolia	10	0.01%	39	0.00%
Martinique	10	0.01%	15	0.00%
Liberia	10	0.01%	39	0.00%
Guinea-Bissau	10	0.01%	111	0.01%
