# Supplementary material for: How the scientific community responded to the COVID-19 pandemic: A subject-level time-trend bibliometric analysis
Source: PLoS One. 2021 Sep 30;16(9):e0258064. doi: 10.1371/journal.pone.0258064 (PMC8483337; doi:10.1371/journal.pone.0258064)
Supplement: S4 Table — (PDF) [file pone.0258064.s004.pdf]

Supplementary Table 4

Research Institute	Country	State/Province	Publication Count
**Harvard Medical School**	United States	Massachusetts	2458
**University Of Toronto**	Canada	Ontario	2227
**Tongji Medical College**	China	Hubei	2003
**Wuhan University**	China	Hubei	1787
**University College London**	United Kingdom	London	1656
**University Of Oxford**	United Kingdom	Oxford	1603
**Stanford University**	United States	California	1363
**University Of Washington**	United States	Washington	1334
**Icahn School Of Medicine At Mount Sinai**	United States	New York	1303
**Mayo Clinic**	United States	Minnesota	1290
**University Of Milan**	Italy	Milan	1253
**Imperial College London**	United Kingdom	London	1141
**King's College London**	United Kingdom	London	1067
**Tehran University Of Medical Sciences**	Iran	Tehran	1023
**Inserm**	France	Paris	997
**The University Of Hong Kong**	China	Hong Kong	990
**University Of Pennsylvania**	United States	Pennsylvania	983
**University Of Health Sciences**	Pakistan	Lahore	979
**Sapienza University of Rome**	Italy	Rome	964
**University Of California, San Francisco**	United States	California	954
