# Supplementary material for: How the scientific community responded to the COVID-19 pandemic: A subject-level time-trend bibliometric analysis
Source: PLoS One. 2021 Sep 30;16(9):e0258064. doi: 10.1371/journal.pone.0258064 (PMC8483337; doi:10.1371/journal.pone.0258064)
Supplement: S5 Table — (PDF) [file pone.0258064.s005.pdf]

Supplementary Table 5

		Publication Count	Publication Share	Citation Count	Citation Share
Europe and Central Asia	United Kingdom	15,691	27.69%	154,236	35.35%
	Italy	13,510	23.84%	126,656	29.03%
	Spain	6,292	11.10%	43,511	9.97%
	France	5,505	9.71%	63,557	14.57%
	Germany	5,461	9.64%	69,720	15.98%
	Turkey	3,377	5.96%	13,287	3.05%
	Switzerland	2,834	5.00%	36,460	8.36%
	Netherlands	2,654	4.68%	38,399	8.80%
	Belgium	1,924	3.40%	18,727	4.29%
	Sweden	1,591	2.81%	16,833	3.86%
	Poland	1,560	2.75%	8,374	1.92%
	Ireland	1,365	2.41%	9,040	2.07%
	Greece	1,356	2.39%	14,984	3.43%
	Portugal	1,257	2.22%	6,286	1.44%
	Austria	1,185	2.09%	16,089	3.69%
	Denmark	1,105	1.95%	14,159	3.25%
	Russian Federation	937	1.65%	12,984	2.98%
	Norway	849	1.50%	7,709	1.77%
	Romania	635	1.12%	2,965	0.68%
	Finland	601	1.06%	4,779	1.10%
	Czech Republic	447	0.79%	2,487	0.57%
	Croatia	358	0.63%	2,197	0.50%
	Hungary	319	0.56%	2,648	0.61%
	Serbia	290	0.51%	801	0.18%
	Slovenia	273	0.48%	1,839	0.42%
	Cyprus	239	0.42%	1,211	0.28%
	Georgia	228	0.40%	661	0.15%
	Ukraine	221	0.39%	797	0.18%
	Slovakia	161	0.28%	469	0.11%
	Lithuania	132	0.23%	945	0.22%
	Bulgaria	129	0.23%	601	0.14%
	Kazakhstan	119	0.21%	400	0.09%
	Luxembourg	112	0.20%	737	0.17%
	Estonia	94	0.17%	1,151	0.26%
	Bosnia And Herzegovina	86	0.15%	143	0.03%
	North Macedonia	53	0.09%	539	0.12%
	Albania	52	0.09%	236	0.05%
	Latvia	44	0.08%	95	0.02%
	Iceland	38	0.07%	555	0.13%
	Azerbaijan	36	0.06%	195	0.04%
	Belarus	31	0.05%	275	0.06%
	Uzbekistan	30	0.05%	31	0.01%
	Armenia	27	0.05%	162	0.04%
	Montenegro	19	0.03%	23	0.01%

Supplementary Table 5

		Publication Count	Publication Share	Citation Count	Citation Share
Europe and Central Asia	Liechtenstein	15	0.03%	39	0.01%
	Gibraltar	12	0.02%	9	0.00%
	Kosovo	11	0.02%	11	0.00%
	Turkmenistan	9	0.02%	39	0.01%
	Monaco	8	0.01%	22	0.01%
	Greenland	7	0.01%	1	0.00%
	Faroe Islands	7	0.01%	119	0.03%
	Moldova	6	0.01%	15	0.00%
	San Marino	5	0.01%	83	0.02%
	Andorra	5	0.01%	20	0.00%
	Tajikistan	2	0.00%	4	0.00%
North America	United States	44,879	91.13%	376,897	92.98%
	Canada	6,428	13.05%	54,622	13.48%
	Bermuda	6	0.01%	26	0.01%
East Asia and Pacific	China	16,485	51.36%	411,151	80.09%
	Australia	5,655	17.62%	53,556	10.43%
	Japan	2,794	8.70%	22,549	4.39%
	South Korea	1,958	6.10%	18,069	3.52%
	Singapore	1,912	5.96%	28,295	5.51%
	Malaysia	1,389	4.33%	4,867	0.95%
	Taiwan	1,276	3.98%	11,111	2.16%
	Indonesia	1,246	3.88%	5,151	1.00%
	New Zealand	841	2.62%	6,897	1.34%
	Thailand	792	2.47%	5,276	1.03%
	Vietnam	549	1.71%	7,019	1.37%
	Philippines	536	1.67%	1,688	0.33%
	Brunei Darussalam	56	0.17%	482	0.09%
	Fiji	36	0.11%	93	0.02%
	Myanmar	27	0.08%	49	0.01%
	Cambodia	27	0.08%	323	0.06%
	Papua New Guinea	13	0.04%	59	0.01%
	Mongolia	10	0.03%	39	0.01%
	Samoa	9	0.03%	9	0.00%
	Solomon Islands	5	0.02%	21	0.00%
	New Caledonia	5	0.02%	8	0.00%
	Vanuatu	4	0.01%	7	0.00%
	French Polynesia	4	0.01%	2	0.00%
	Tonga	3	0.01%	0	0.00%
	Timor-Leste	3	0.01%	2	0.00%
	Guam	2	0.01%	11	0.00%
South Asia	India	11,846	78.77%	45,356	76.37%
	Pakistan	1,999	13.29%	9,391	15.81%
	Bangladesh	1,034	6.88%	5,646	9.51%
	Nepal	432	2.87%	2,972	5.00%

Supplementary Table 5

		Publication Count	Publication Share	Citation Count	Citation Share
South Asia	Sri Lanka	146	0.97%	796	1.34%
	Afghanistan	53	0.35%	107	0.18%
	Bhutan	14	0.09%	22	0.04%
	Maldives	12	0.08%	161	0.27%
Middle East and North Africa	Iran	4,142	31.81%	20,598	33.37%
	Saudi Arabia	2,809	21.57%	15,421	24.98%
	Egypt	1,550	11.90%	6,585	10.67%
	Israel	1,461	11.22%	9,939	16.10%
	United Arab Emirates	819	6.29%	4,219	6.84%
	Jordan	583	4.48%	2,331	3.78%
	Qatar	553	4.25%	2,350	3.81%
	Morocco	496	3.81%	1,544	2.50%
	Lebanon	463	3.56%	2,467	4.00%
	Iraq	412	3.16%	1,530	2.48%
	Oman	273	2.10%	2,634	4.27%
	Tunisia	232	1.78%	1,156	1.87%
	Kuwait	226	1.74%	1,487	2.41%
	Algeria	141	1.08%	383	0.62%
	Malta	113	0.87%	381	0.62%
	Bahrain	110	0.84%	252	0.41%
	Palestine	99	0.76%	207	0.34%
	Yemen	86	0.66%	295	0.48%
	Libya	73	0.56%	153	0.25%
	Syria	40	0.31%	68	0.11%
	Djibouti	3	0.02%	3	0.00%
Sub-Saharan Africa	South Africa	2,058	41.96%	9,280	46.86%
	Nigeria	1,034	21.08%	3,062	15.46%
	Ethiopia	471	9.60%	1,170	5.91%
	Kenya	347	7.07%	1,033	5.22%
	Ghana	320	6.52%	804	4.06%
	Uganda	218	4.44%	776	3.92%
	Cameroon	160	3.26%	714	3.61%
	Sudan	139	2.83%	619	3.13%
	Tanzania	118	2.41%	539	2.72%
	Zimbabwe	103	2.10%	418	2.11%
	Senegal	91	1.86%	923	4.66%
	Democratic Republic Of The Congo	87	1.77%	300	1.51%
	Zambia	79	1.61%	270	1.36%
	Malawi	75	1.53%	265	1.34%
	Mozambique	65	1.33%	926	4.68%
	Rwanda	55	1.12%	159	0.80%
	Mali	40	0.82%	429	2.17%
	Congo	39	0.80%	1,279	6.46%
	Botswana	39	0.80%	119	0.60%

Supplementary Table 5

		Publication Count	Publication Share	Citation Count	Citation Share
Sub-Saharan Africa	Burkina Faso	36	0.73%	49	0.25%
	Sierra Leone	35	0.71%	59	0.30%
	Mauritius	33	0.67%	384	1.94%
	Benin	33	0.67%	93	0.47%
	Gabon	31	0.63%	115	0.58%
	Guinea	27	0.55%	117	0.59%
	Madagascar	25	0.51%	58	0.29%
	Cote D'Ivoire	18	0.37%	386	1.95%
	Namibia	17	0.35%	48	0.24%
	Somalia	15	0.31%	61	0.31%
	Togo	13	0.27%	26	0.13%
	Niger	13	0.27%	4	0.02%
	Liberia	10	0.20%	39	0.20%
	Guinea-Bissau	10	0.20%	111	0.56%
	Lesotho	9	0.18%	42	0.21%
	Mauritania	6	0.12%	12	0.06%
	Eritrea	6	0.12%	13	0.07%
	Burundi	6	0.12%	3	0.02%
	Chad	5	0.10%	5	0.03%
	Central African Republic	4	0.08%	53	0.27%
	Seychelles	3	0.06%	5	0.03%
	Angola	3	0.06%	0	0.00%
	Equatorial Guinea	1	0.02%	2	0.01%
	Comoros	1	0.02%	3	0.02%
Latin America and Caribbean	Brazil	4,636	53.66%	22,635	55.48%
	Mexico	1,298	15.02%	7,287	17.86%
	Colombia	780	9.03%	5,478	13.43%
	Argentina	631	7.30%	3,821	9.36%
	Chile	627	7.26%	2,776	6.80%
	Peru	509	5.89%	3,181	7.80%
	Venezuela	282	3.26%	2,004	4.91%
	Ecuador	270	3.13%	1,679	4.12%
	Uruguay	131	1.52%	424	1.04%
	Cuba	101	1.17%	148	0.36%
	Jamaica	73	0.85%	185	0.45%
	Bolivia	68	0.79%	1,220	2.99%
	Costa Rica	64	0.74%	232	0.57%
	Panama	60	0.69%	1,063	2.61%
	Nicaragua	48	0.56%	69	0.17%
	Honduras	44	0.51%	1,353	3.32%
	Paraguay	43	0.50%	716	1.75%
	Puerto Rico	42	0.49%	368	0.90%
	Guatemala	35	0.41%	157	0.38%
	Dominican Republic	31	0.36%	68	0.17%

Supplementary Table 5

		Publication Count	Publication Share	Citation Count	Citation Share
Latin America and Caribbean	Trinidad And Tobago	28	0.32%	69	0.17%
	Barbados	24	0.28%	29	0.07%
	Grenada	19	0.22%	54	0.13%
	El Salvador	17	0.20%	37	0.09%
	Aruba	12	0.14%	114	0.28%
	Haiti	11	0.13%	19	0.05%
	Curacao	8	0.09%	97	0.24%
	Guyana	5	0.06%	46	0.11%
	Dominica	5	0.06%	8	0.02%
	Antigua And Barbuda	4	0.05%	2	0.00%
	Belize	3	0.03%	2	0.00%
	Suriname	1	0.01%	21	0.05%
