# Supplementary material for: How the scientific community responded to the COVID-19 pandemic: A subject-level time-trend bibliometric analysis
Source: PLoS One. 2021 Sep 30;16(9):e0258064. doi: 10.1371/journal.pone.0258064 (PMC8483337; doi:10.1371/journal.pone.0258064)
Supplement: S6 Table — (PDF) [file pone.0258064.s006.pdf]

## Supplementary Table 6

| | | Publication Count | Publication Share | Citation Count | Citation Share |
|---|---|---|---|---|---|
| High Income | United States | 44,879 | 42.33% | 376,897 | 47.91% |
| | United Kingdom | 15,691 | 14.80% | 154,236 | 19.61% |
| | Italy | 13,510 | 12.74% | 126,656 | 16.10% |
| | Canada | 6,428 | 6.06% | 54,622 | 6.94% |
| | Spain | 6,292 | 5.93% | 43,511 | 5.53% |
| | Australia | 5,655 | 5.33% | 53,556 | 6.81% |
| | France | 5,505 | 5.19% | 63,557 | 8.08% |
| | Germany | 5,461 | 5.15% | 69,720 | 8.86% |
| | Switzerland | 2,834 | 2.67% | 36,460 | 4.63% |
| | Saudi Arabia | 2,809 | 2.65% | 15,421 | 1.96% |
| | Japan | 2,794 | 2.64% | 22,549 | 2.87% |
| | Netherlands | 2,654 | 2.50% | 38,399 | 4.88% |
| | South Korea | 1,958 | 1.85% | 18,069 | 2.30% |
| | Belgium | 1,924 | 1.81% | 18,727 | 2.38% |
| | Singapore | 1,912 | 1.80% | 28,295 | 3.60% |
| | Sweden | 1,591 | 1.50% | 16,833 | 2.14% |
| | Poland | 1,560 | 1.47% | 8,374 | 1.06% |
| | Israel | 1,461 | 1.38% | 9,939 | 1.26% |
| | Ireland | 1,365 | 1.29% | 9,040 | 1.15% |
| | Greece | 1,356 | 1.28% | 14,984 | 1.90% |
| | Taiwan | 1,276 | 1.20% | 11,111 | 1.41% |
| | Portugal | 1,257 | 1.19% | 6,286 | 0.80% |
| | Austria | 1,185 | 1.12% | 16,089 | 2.05% |
| | Denmark | 1,105 | 1.04% | 14,159 | 1.80% |
| | Norway | 849 | 0.80% | 7,709 | 0.98% |
| | New Zealand | 841 | 0.79% | 6,897 | 0.88% |
| | United Arab Emirates | 819 | 0.77% | 4,219 | 0.54% |
| | Romania | 635 | 0.60% | 2,965 | 0.38% |
| | Chile | 627 | 0.59% | 2,776 | 0.35% |
| | Finland | 601 | 0.57% | 4,779 | 0.61% |
| | Qatar | 553 | 0.52% | 2,350 | 0.30% |
| | Czech Republic | 447 | 0.42% | 2,487 | 0.32% |
| | Croatia | 358 | 0.34% | 2,197 | 0.28% |
| | Hungary | 319 | 0.30% | 2,648 | 0.34% |
| | Slovenia | 273 | 0.26% | 1,839 | 0.23% |
| | Oman | 273 | 0.26% | 2,634 | 0.33% |
| | Cyprus | 239 | 0.23% | 1,211 | 0.15% |
| | Kuwait | 226 | 0.21% | 1,487 | 0.19% |
| | Slovakia | 161 | 0.15% | 469 | 0.06% |
| | Lithuania | 132 | 0.12% | 945 | 0.12% |
| | Uruguay | 131 | 0.12% | 424 | 0.05% |
| | Malta | 113 | 0.11% | 381 | 0.05% |
| | Luxembourg | 112 | 0.11% | 737 | 0.09% |
| | Bahrain | 110 | 0.10% | 252 | 0.03% |

# Supplementary Table 6

| | | Publication Count | Publication Share | Citation Count | Citation Share |
|---|---|---|---|---|---|
| High Income | Estonia | 94 | 0.09% | 1,151 | 0.15% |
| | Panama | 60 | 0.06% | 1,063 | 0.14% |
| | Brunei Darussalam | 56 | 0.05% | 482 | 0.06% |
| | Latvia | 44 | 0.04% | 95 | 0.01% |
| | Puerto Rico | 42 | 0.04% | 368 | 0.05% |
| | Iceland | 38 | 0.04% | 555 | 0.07% |
| | Mauritius | 33 | 0.03% | 384 | 0.05% |
| | Trinidad And Tobago | 28 | 0.03% | 69 | 0.01% |
| | Barbados | 24 | 0.02% | 29 | 0.00% |
| | Liechtenstein | 15 | 0.01% | 39 | 0.00% |
| | Gibraltar | 12 | 0.01% | 9 | 0.00% |
| | Aruba | 12 | 0.01% | 114 | 0.01% |
| | Monaco | 8 | 0.01% | 22 | 0.00% |
| | Curacao | 8 | 0.01% | 97 | 0.01% |
| | Greenland | 7 | 0.01% | 1 | 0.00% |
| | Faroe Islands | 7 | 0.01% | 119 | 0.02% |
| | Bermuda | 6 | 0.01% | 26 | 0.00% |
| | San Marino | 5 | 0.00% | 83 | 0.01% |
| | New Caledonia | 5 | 0.00% | 8 | 0.00% |
| | Andorra | 5 | 0.00% | 20 | 0.00% |
| | French Polynesia | 4 | 0.00% | 2 | 0.00% |
| | Antigua And Barbuda | 4 | 0.00% | 2 | 0.00% |
| | Seychelles | 3 | 0.00% | 5 | 0.00% |
| | Guam | 2 | 0.00% | 11 | 0.00% |
| Upper-middle Income | China | 16,485 | 42.73% | 411,151 | 81.70% |
| | Brazil | 4,636 | 12.02% | 22,635 | 4.50% |
| | Iran | 4,142 | 10.74% | 20,598 | 4.09% |
| | Turkey | 3,377 | 8.75% | 13,287 | 2.64% |
| | South Africa | 2,058 | 5.33% | 9,280 | 1.84% |
| | Malaysia | 1,389 | 3.60% | 4,867 | 0.97% |
| | Mexico | 1,298 | 3.36% | 7,287 | 1.45% |
| | Indonesia | 1,246 | 3.23% | 5,151 | 1.02% |
| | Russian Federation | 937 | 2.43% | 12,984 | 2.58% |
| | Thailand | 792 | 2.05% | 5,276 | 1.05% |
| | Colombia | 780 | 2.02% | 5,478 | 1.09% |
| | Argentina | 631 | 1.64% | 3,821 | 0.76% |
| | Jordan | 583 | 1.51% | 2,331 | 0.46% |
| | Peru | 509 | 1.32% | 3,181 | 0.63% |
| | Lebanon | 463 | 1.20% | 2,467 | 0.49% |
| | Iraq | 412 | 1.07% | 1,530 | 0.30% |
| | Serbia | 290 | 0.75% | 801 | 0.16% |
| | Venezuela | 282 | 0.73% | 2,004 | 0.40% |
| | Ecuador | 270 | 0.70% | 1,679 | 0.33% |
| | Georgia | 228 | 0.59% | 661 | 0.13% |

# Supplementary Table 6

| | | Publication Count | Publication Share | Citation Count | Citation Share |
|---|---|---|---|---|---|
| Upper-middle Income | Bulgaria | 129 | 0.33% | 601 | 0.12% |
| | Kazakhstan | 119 | 0.31% | 400 | 0.08% |
| | Cuba | 101 | 0.26% | 148 | 0.03% |
| | Bosnia And Herzegovina | 86 | 0.22% | 143 | 0.03% |
| | Libya | 73 | 0.19% | 153 | 0.03% |
| | Jamaica | 73 | 0.19% | 185 | 0.04% |
| | Costa Rica | 64 | 0.17% | 232 | 0.05% |
| | North Macedonia | 53 | 0.14% | 539 | 0.11% |
| | Albania | 52 | 0.13% | 236 | 0.05% |
| | Paraguay | 43 | 0.11% | 716 | 0.14% |
| | Botswana | 39 | 0.10% | 119 | 0.02% |
| | Fiji | 36 | 0.09% | 93 | 0.02% |
| | Azerbaijan | 36 | 0.09% | 195 | 0.04% |
| | Guatemala | 35 | 0.09% | 157 | 0.03% |
| | Gabon | 31 | 0.08% | 115 | 0.02% |
| | Dominican Republic | 31 | 0.08% | 68 | 0.01% |
| | Belarus | 31 | 0.08% | 275 | 0.05% |
| | Armenia | 27 | 0.07% | 162 | 0.03% |
| | Montenegro | 19 | 0.05% | 23 | 0.00% |
| | Grenada | 19 | 0.05% | 54 | 0.01% |
| | Namibia | 17 | 0.04% | 48 | 0.01% |
| | Maldives | 12 | 0.03% | 161 | 0.03% |
| | Kosovo | 11 | 0.03% | 11 | 0.00% |
| | Turkmenistan | 9 | 0.02% | 39 | 0.01% |
| | Samoa | 9 | 0.02% | 9 | 0.00% |
| | Guyana | 5 | 0.01% | 46 | 0.01% |
| | Dominica | 5 | 0.01% | 8 | 0.00% |
| | Tonga | 3 | 0.01% | 0 | 0.00% |
| | Belize | 3 | 0.01% | 2 | 0.00% |
| | Suriname | 1 | 0.00% | 21 | 0.00% |
| | Equatorial Guinea | 1 | 0.00% | 2 | 0.00% |
| Lower-middle Income | India | 11,846 | 58.38% | 45,356 | 55.19% |
| | Pakistan | 1,999 | 9.85% | 9,391 | 11.43% |
| | Egypt | 1,550 | 7.64% | 6,585 | 8.01% |
| | Nigeria | 1,034 | 5.10% | 3,062 | 3.73% |
| | Bangladesh | 1,034 | 5.10% | 5,646 | 6.87% |
| | Vietnam | 549 | 2.71% | 7,019 | 8.54% |
| | Philippines | 536 | 2.64% | 1,688 | 2.05% |
| | Morocco | 496 | 2.44% | 1,544 | 1.88% |
| | Nepal | 432 | 2.13% | 2,972 | 3.62% |
| | Kenya | 347 | 1.71% | 1,033 | 1.26% |
| | Ghana | 320 | 1.58% | 804 | 0.98% |
| | Tunisia | 232 | 1.14% | 1,156 | 1.41% |
| | Ukraine | 221 | 1.09% | 797 | 0.97% |

# Supplementary Table 6

| | | Publication Count | Publication Share | Citation Count | Citation Share |
|---|---|---|---|---|---|
| Lower-middle Income | Cameroon | 160 | 0.79% | 714 | 0.87% |
| | Sri Lanka | 146 | 0.72% | 796 | 0.97% |
| | Algeria | 141 | 0.69% | 383 | 0.47% |
| | Tanzania | 118 | 0.58% | 539 | 0.66% |
| | Zimbabwe | 103 | 0.51% | 418 | 0.51% |
| | Palestine | 99 | 0.49% | 207 | 0.25% |
| | Senegal | 91 | 0.45% | 923 | 1.12% |
| | Zambia | 79 | 0.39% | 270 | 0.33% |
| | Bolivia | 68 | 0.34% | 1,220 | 1.48% |
| | Nicaragua | 48 | 0.24% | 69 | 0.08% |
| | Honduras | 44 | 0.22% | 1,353 | 1.65% |
| | Congo | 39 | 0.19% | 1,279 | 1.56% |
| | Benin | 33 | 0.16% | 93 | 0.11% |
| | Uzbekistan | 30 | 0.15% | 31 | 0.04% |
| | Myanmar | 27 | 0.13% | 49 | 0.06% |
| | Cambodia | 27 | 0.13% | 323 | 0.39% |
| | Cote D'Ivoire | 18 | 0.09% | 386 | 0.47% |
| | El Salvador | 17 | 0.08% | 37 | 0.05% |
| | Bhutan | 14 | 0.07% | 22 | 0.03% |
| | Papua New Guinea | 13 | 0.06% | 59 | 0.07% |
| | Mongolia | 10 | 0.05% | 39 | 0.05% |
| | Lesotho | 9 | 0.04% | 42 | 0.05% |
| | Moldova | 6 | 0.03% | 15 | 0.02% |
| | Mauritania | 6 | 0.03% | 12 | 0.01% |
| | Solomon Islands | 5 | 0.02% | 21 | 0.03% |
| | Vanuatu | 4 | 0.02% | 7 | 0.01% |
| | Timor-Leste | 3 | 0.01% | 2 | 0.00% |
| | Djibouti | 3 | 0.01% | 3 | 0.00% |
| | Angola | 3 | 0.01% | 0 | 0.00% |
| | Comoros | 1 | 0.00% | 3 | 0.00% |
| Low Income | Ethiopia | 471 | 33.88% | 1,170 | 24.14% |
| | Uganda | 218 | 15.68% | 776 | 16.01% |
| | Sudan | 139 | 10.00% | 619 | 12.77% |
| | Democratic Republic Of The Congo | 87 | 6.26% | 300 | 6.19% |
| | Yemen | 86 | 6.19% | 295 | 6.09% |
| | Malawi | 75 | 5.40% | 265 | 5.47% |
| | Mozambique | 65 | 4.68% | 926 | 19.11% |
| | Rwanda | 55 | 3.96% | 159 | 3.28% |
| | Afghanistan | 53 | 3.81% | 107 | 2.21% |
| | Syria | 40 | 2.88% | 68 | 1.40% |
| | Mali | 40 | 2.88% | 429 | 8.85% |
| | Burkina Faso | 36 | 2.59% | 49 | 1.01% |
| | Sierra Leone | 35 | 2.52% | 59 | 1.22% |
| | Guinea | 27 | 1.94% | 117 | 2.41% |

# Supplementary Table 6

| | | Publication Count | Publication Share | Citation Count | Citation Share |
|---|---|---|---|---|---|
| Low Income | Madagascar | 25 | 1.80% | 58 | 1.20% |
| | Somalia | 15 | 1.08% | 61 | 1.26% |
| | Togo | 13 | 0.94% | 26 | 0.54% |
| | Niger | 13 | 0.94% | 4 | 0.08% |
| | Haiti | 11 | 0.79% | 19 | 0.39% |
| | Liberia | 10 | 0.72% | 39 | 0.80% |
| | Guinea-Bissau | 10 | 0.72% | 111 | 2.29% |
| | Eritrea | 6 | 0.43% | 13 | 0.27% |
| | Burundi | 6 | 0.43% | 3 | 0.06% |
| | Chad | 5 | 0.36% | 5 | 0.10% |
| | Central African Republic | 4 | 0.29% | 53 | 1.09% |
| | Tajikistan | 2 | 0.14% | 4 | 0.08% |
