# Supplementary material for: How the scientific community responded to the COVID-19 pandemic: A subject-level time-trend bibliometric analysis
Source: PLoS One. 2021 Sep 30;16(9):e0258064. doi: 10.1371/journal.pone.0258064 (PMC8483337; doi:10.1371/journal.pone.0258064)
Supplement: S7 Table — (PDF) [file pone.0258064.s007.pdf]

Supplementary Table 7

	Annual Publications	COVID-19 Share
Bangladesh	3,135	32.98%
Saudi Arabia	10,898	25.78%
Ireland	7,174	19.03%
Italy	71,240	18.96%
Nigeria	5,602	18.46%
Singapore	11,459	16.69%
United Kingdom	97,681	16.06%
South Africa	13,009	15.82%
Pakistan	12,904	15.49%
Switzerland	21,379	13.26%
Greece	10,907	12.43%
Belgium	15,688	12.26%
Israel	12,235	11.94%
Egypt	13,327	11.63%
Spain	54,537	11.54%
Canada	59,968	10.72%
United States	422,808	10.61%
Australia	53,610	10.55%
Turkey	33,536	10.07%
Austria	12,362	9.59%
Portugal	14,295	8.79%
India	135,788	8.72%
Netherlands	30,457	8.71%
Iran	48,306	8.57%
France	66,352	8.30%
Mexico	16,346	7.94%
Denmark	13,979	7.90%
Sweden	20,421	7.79%
Brazil	60,148	7.71%
Malaysia	23,661	5.87%
Germany	104,396	5.23%
Indonesia	26,948	4.62%
Poland	35,663	4.37%
China	528,263	3.12%
South Korea	66,376	2.95%
Japan	98,793	2.83%
