# Supplementary material for: How the scientific community responded to the COVID-19 pandemic: A subject-level time-trend bibliometric analysis
Source: PLoS One. 2021 Sep 30;16(9):e0258064. doi: 10.1371/journal.pone.0258064 (PMC8483337; doi:10.1371/journal.pone.0258064)
Supplement: S8 Table — (PDF) [file pone.0258064.s008.pdf]

Supplementary Table 8

	Degree	Publications	Degree to Publications Ratio	Degree Centrality
United States	31,668	44,879	1.42	92.0%
United Kingdom	21,517	15,691	0.73	82.6%
Italy	15,529	13,510	0.87	76.1%
China	11,803	16,485	1.40	70.9%
Germany	10,967	5,461	0.50	65.3%
Canada	10,009	6,428	0.64	71.8%
Australia	9,969	5,655	0.57	70.9%
Spain	9,819	6,292	0.64	71.4%
France	9,465	5,505	0.58	74.6%
India	8,571	11,846	1.38	74.6%
Switzerland	7,606	2,834	0.37	74.6%
Netherlands	7,326	2,654	0.36	67.1%
Brazil	6,249	4,636	0.74	68.5%
Belgium	5,320	1,924	0.36	60.1%
Sweden	4,775	1,591	0.33	61.5%
South Africa	4,597	2,058	0.45	70.0%
Saudi Arabia	4,544	2,809	0.62	59.6%
Japan	4,218	2,794	0.66	67.1%
Turkey	3,899	3,377	0.87	60.6%
Iran	3,752	4,142	1.10	62.0%
Austria	3,617	1,185	0.33	54.9%
Portugal	3,576	1,257	0.35	58.2%
Greece	3,422	1,356	0.40	51.2%
Singapore	3,420	1,912	0.56	57.3%
Poland	3,294	1,560	0.47	59.6%
Egypt	3,273	1,550	0.47	62.9%
Denmark	3,253	1,105	0.34	52.6%
Pakistan	3,043	1,999	0.66	64.8%
Ireland	2,997	1,365	0.46	57.7%
South Korea	2,788	1,958	0.70	52.6%
Mexico	2,700	1,298	0.48	61.0%
Malaysia	2,609	1,389	0.53	62.4%
Israel	2,453	1,461	0.60	49.8%
Nigeria	2,272	1,034	0.46	64.8%
Taiwan	1,840	1,276	0.69	49.3%
Bangladesh	1,801	1,034	0.57	58.7%
Indonesia	1,444	1,246	0.86	58.7%
