# Supplementary material for: How the scientific community responded to the COVID-19 pandemic: A subject-level time-trend bibliometric analysis
Source: PLoS One. 2021 Sep 30;16(9):e0258064. doi: 10.1371/journal.pone.0258064 (PMC8483337; doi:10.1371/journal.pone.0258064)
Supplement: S9 Table — (PDF) [file pone.0258064.s009.pdf]

Supplementary Table 9

	Open-Access Percent	Open-Access Publications
Sweden	86.23%	1,102
Netherlands	82.67%	1,836
Brazil	82.03%	3,067
Switzerland	81.67%	1,912
Austria	81.50%	806
Germany	80.43%	3,460
Belgium	80.01%	1,241
Japan	79.99%	1,671
South Korea	79.96%	1,325
Poland	79.93%	980
Taiwan	79.48%	821
China	77.99%	10,011
Denmark	77.96%	711
Saudi Arabia	77.38%	1,563
Portugal	77.21%	752
United Kingdom	76.99%	10,213
Singapore	76.34%	1,171
Italy	76.00%	8,114
Egypt	75.99%	918
Canada	75.78%	3,887
Greece	74.28%	800
Bangladesh	74.11%	541
Australia	73.95%	3,529
Mexico	73.78%	740
Turkey	73.20%	1,863
France	73.14%	3,241
United States	73.02%	23,500
Ireland	73.00%	803
South Africa	72.65%	1,113
Israel	72.55%	806
Spain	72.50%	3,656
Pakistan	71.63%	1,035
Malaysia	69.77%	771
Iran	68.44%	2,067
Nigeria	68.25%	531
India	67.31%	5,843
Indonesia	64.03%	680
