# Supplementary material for: How the scientific community responded to the COVID-19 pandemic: A subject-level time-trend bibliometric analysis
Source: PLoS One. 2021 Sep 30;16(9):e0258064. doi: 10.1371/journal.pone.0258064 (PMC8483337; doi:10.1371/journal.pone.0258064)
Supplement: S10 Table — (PDF) [file pone.0258064.s010.pdf]

Supplementary Table 10

Journal	Publication Count
International Journal Of Environmental Research And Public Health	1,556
Plos One	1,243
Journal Of Medical Virology	1,004
Frontiers In Psychology	974
BMJ (Clinical Research Ed.)	869
Frontiers In Public Health	737
Frontiers In Medicine	723
Cureus	707
The BMJ	686
Disaster Medicine And Public Health Preparedness	615
Sustainability (Switzerland)	569
International Journal Of Infectious Diseases	567
Frontiers In Psychiatry	558
Scientific Reports	541
Clinical Infectious Diseases	526
JAMA	502
The Lancet	490
Infection Control And Hospital Epidemiology	484
Medical Hypotheses	441
Journal Of Medical Internet Research	438
Science Of The Total Environment	427
New England Journal Of Medicine	426
Journal Of Infection	419
Nature	407
Journal Of Clinical Medicine	395
Dermatologic Therapy	384
Eclinicalmedicine	368
Frontiers In Pharmacology	365
Frontiers In Immunology	354
Annals Of The Rheumatic Diseases	341
