# Supplementary material for: How the scientific community responded to the COVID-19 pandemic: A subject-level time-trend bibliometric analysis
Source: PLoS One. 2021 Sep 30;16(9):e0258064. doi: 10.1371/journal.pone.0258064 (PMC8483337; doi:10.1371/journal.pone.0258064)
Supplement: S11 Table — (PDF) [file pone.0258064.s011.pdf]

Supplementary Table 11

Journal	Citation Count
New England Journal Of Medicine	67,015
The Lancet	67,006
JAMA	43,635
Nature	22,644
Journal Of Medical Virology	20,031
Science	18,210
The Lancet Infectious Diseases	15,488
Cell	14,464
Radiology	12,509
The Lancet Respiratory Medicine	12,458
Clinical Infectious Diseases	12,211
The BMJ	11,840
Science Of The Total Environment	11,161
Nature Medicine	10,621
International Journal Of Infectious Diseases	9,990
Journal Of Infection	9,096
International Journal Of Environmental Research And Public Health	8,938
International Journal Of Antimicrobial Agents	7,993
Morbidity And Mortality Weekly Report	7,516
Eurosurveillance	7,035
Proceedings Of The National Academy Of Sciences (PNAS)	6,903
Annals Of Internal Medicine	6,860
Journal Of Thrombosis And Haemostasis	6,334
Intensive Care Medicine	6,049
Emerging Microbes And Infections	6,004
The Lancet Psychiatry	5,956
Brain, Behavior, And Immunity	5,936
JAMA Cardiology	5,833
Emerging Infectious Diseases	5,755
Psychiatry Research	5,318
