# Supplementary material for: How the scientific community responded to the COVID-19 pandemic: A subject-level time-trend bibliometric analysis
Source: PLoS One. 2021 Sep 30;16(9):e0258064. doi: 10.1371/journal.pone.0258064 (PMC8483337; doi:10.1371/journal.pone.0258064)
Supplement: S12 Table — (PDF) [file pone.0258064.s012.pdf]

Supplementary Table 12

		Publication Count	Publication Share	Citation Count	Citation Share
Health Sciences	International Journal Of Environmental Research And Public Health	1,327	1.60%	8,922	1.04%
	Journal Of Medical Virology	950	1.15%	20,008	2.33%
	BMJ (Clinical Research Ed.)	782	0.94%	3,944	0.46%
	The BMJ	686	0.83%	11,840	1.38%
	International Journal Of Infectious Diseases	567	0.68%	9,990	1.16%
	The Lancet	490	0.59%	67,006	7.79%
	Frontiers In Public Health	460	0.56%	1,782	0.21%
	JAMA	432	0.52%	42,740	4.97%
	Medical Hypotheses	421	0.51%	1,653	0.19%
	Journal Of Infection	419	0.51%	9,096	1.06%
Life Sciences	Plos One	1,210	4.48%	4,603	1.64%
	Journal Of Medical Virology	950	3.51%	20,008	7.13%
	Frontiers In Immunology	335	1.24%	2,445	0.87%
	Journal Of Biomolecular Structure And Dynamics	306	1.13%	3,976	1.42%
	Annals Of The Rheumatic Diseases	245	0.91%	2,499	0.89%
	Psychiatry Research	244	0.90%	5,293	1.89%
	Nature Communications	241	0.89%	3,869	1.38%
	International Journal Of Research In Pharmaceutical Sciences	241	0.89%	187	0.07%
	American Journal Of Tropical Medicine And Hygiene	216	0.80%	989	0.35%
	Journal Of Clinical Virology	194	0.72%	3,633	1.29%
	Viruses	192	0.71%	2,629	0.94%
	International Journal Of Molecular Sciences	191	0.71%	1,320	0.47%
	Frontiers In Pharmacology	188	0.70%	488	0.17%
	Nature Medicine	153	0.57%	10,524	3.75%
	Vaccines	148	0.55%	476	0.17%
	Eurosurveillance	146	0.54%	7,035	2.51%
	Environmental Research	146	0.54%	1,338	0.48%
	Emerging Microbes And Infections	141	0.52%	6,004	2.14%
	Frontiers In Neurology	138	0.51%	504	0.18%
	Diabetes Research And Clinical Practice	137	0.51%	1,407	0.50%
Physical Sciences	International Journal Of Environmental Research And Public Health	1,327	8.76%	8,922	10.40%
	Sustainability (Switzerland)	569	3.76%	1,569	1.83%
	Science Of The Total Environment	427	2.82%	11,161	13.00%
	Nature Communications	241	1.59%	3,869	4.51%
	Chaos, Solitons And Fractals	231	1.53%	2,913	3.39%
	International Journal Of Molecular Sciences	191	1.26%	1,320	1.54%
	Ieee Access	189	1.25%	849	0.99%
	Journal Of Chemical Education	186	1.23%	355	0.41%
	Morbidity And Mortality Weekly Report	184	1.22%	7,516	8.76%
	Environmental Research	146	0.96%	1,338	1.56%
	Journal Of Cleaner Production	110	0.73%	391	0.46%
	Indian Journal Of Forensic Medicine And Toxicology	104	0.69%	8	0.01%
	Environmental Science And Pollution Research	91	0.60%	116	0.14%
	Applied Sciences (Switzerland)	88	0.58%	162	0.19%
	Infectious Disease Modelling	84	0.55%	1,034	1.20%
	Computers, Materials And Continua	83	0.55%	169	0.20%
Social Sciences	Sustainability (Switzerland)	569	2.80%	1,569	2.21%
	Frontiers In Psychology	494	2.43%	1,152	1.62%
	Asian Journal Of Psychiatry	237	1.17%	3,449	4.85%
	Journal Of Chemical Education	186	0.92%	355	0.50%
	Morbidity And Mortality Weekly Report	184	0.91%	7,516	10.57%
	Psychological Trauma: Theory, Research, Practice, And Policy	156	0.77%	990	1.39%
	Economic And Political Weekly	134	0.66%	104	0.15%
	Journal Of Affective Disorders	124	0.61%	1,160	1.63%
	Personality And Individual Differences	119	0.59%	344	0.48%
	Journal Of Cleaner Production	110	0.54%	391	0.55%
	Finance Research Letters	110	0.54%	1,116	1.57%
	Indian Journal Of Forensic Medicine And Toxicology	104	0.51%	8	0.01%
	World Development	102	0.50%	309	0.43%
