# Supplementary figures and images for: How the scientific community responded to the COVID-19 pandemic: A subject-level time-trend bibliometric analysis
Source: PLoS One. 2021 Sep 30;16(9):e0258064. doi: 10.1371/journal.pone.0258064 (PMC8483337; doi:10.1371/journal.pone.0258064)

Supplementary Figure 1

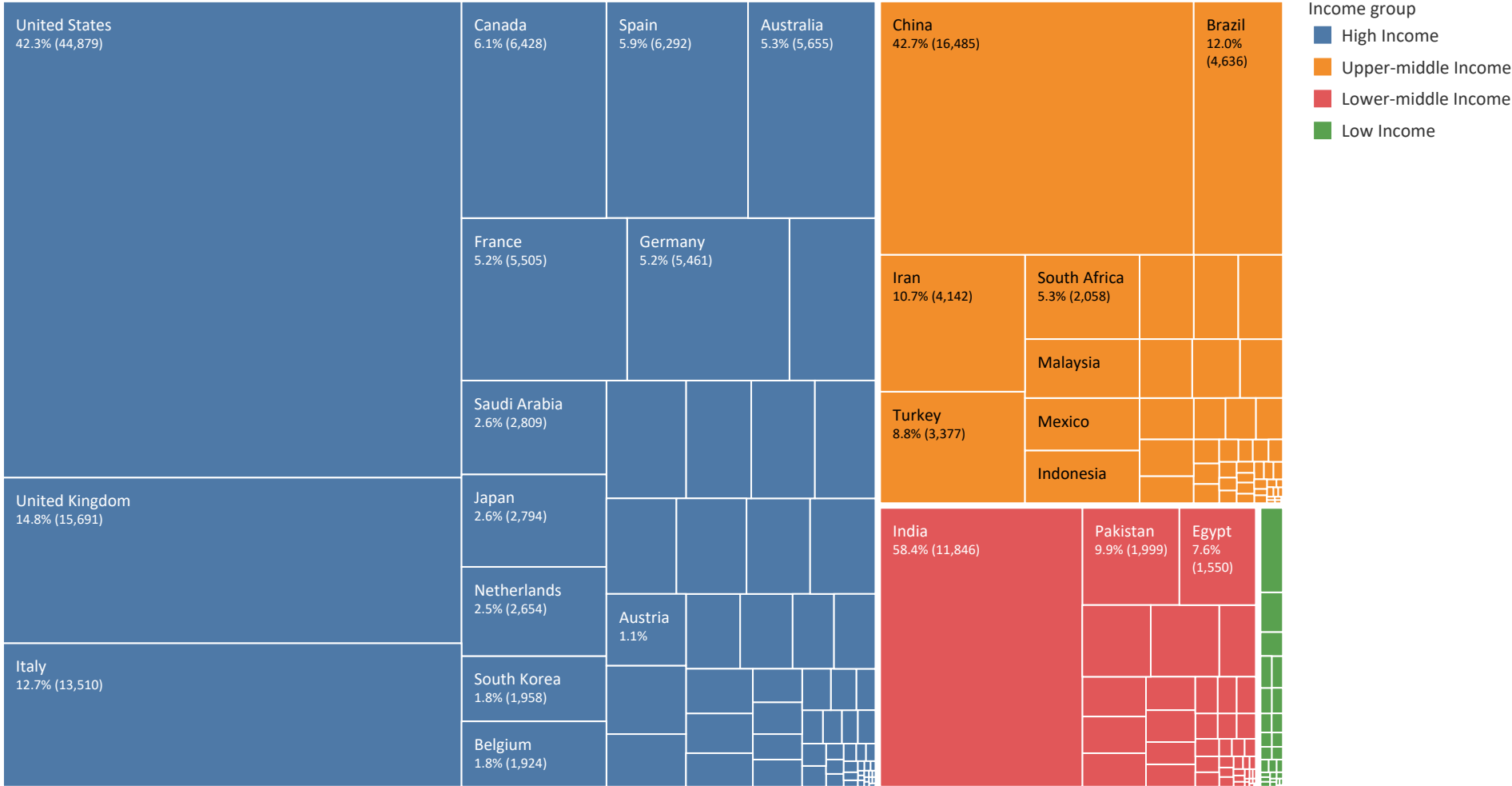

Supplement: S1 Fig — (PDF) [file pone.0258064.s013.pdf]

**Supplementary Figure 3**

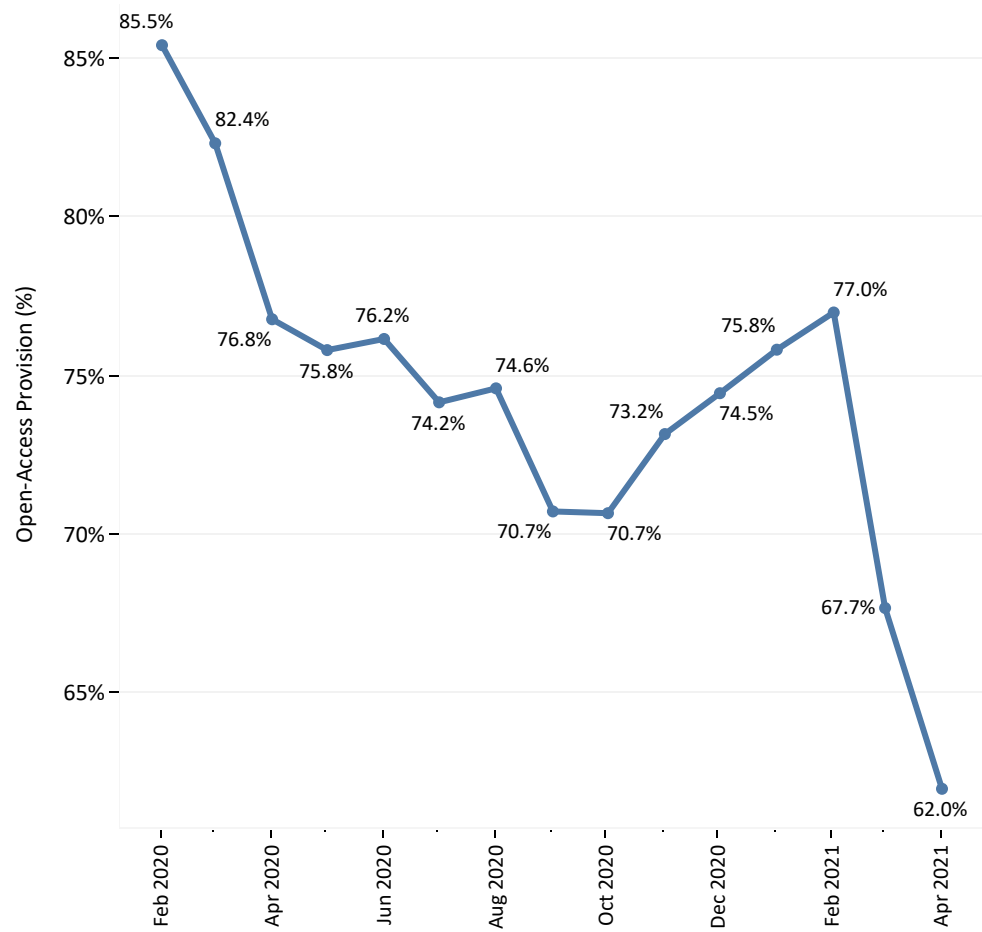

Supplement: S3 Fig — (PDF) [file pone.0258064.s015.pdf]
