# Supplementary material for: How the scientific community responded to the COVID-19 pandemic: A subject-level time-trend bibliometric analysis
Source: PLoS One. 2021 Sep 30;16(9):e0258064. doi: 10.1371/journal.pone.0258064 (PMC8483337; doi:10.1371/journal.pone.0258064)
Supplement: S2 Fig — (PDF) [file pone.0258064.s014.pdf]

Supplementary Figure 2

	National	Bi-National	Multi-National
Switzerland	25.3% (716)	28.8% (817)	45.9% (1,301)
Sweden	25.6% (407)	23.9% (380)	50.5% (804)
Denmark	29.0% (320)	24.6% (272)	46.4% (513)
Austria	29.5% (350)	24.9% (295)	45.6% (540)
Netherlands	31.9% (846)	23.4% (621)	44.7% (1,187)
Belgium	32.3% (622)	22.2% (427)	45.5% (875)
Saudi Arabia	35.3% (991)	31.4% (882)	33.3% (936)
Egypt	36.5% (566)	28.7% (445)	34.8% (539)
Malaysia	37.5% (521)	27.4% (380)	35.1% (488)
Bangladesh	38.2% (395)	27.6% (285)	34.2% (354)
South Africa	38.8% (798)	24.1% (497)	37.1% (763)
Australia	38.9% (2,197)	27.6% (1,560)	33.6% (1,898)
Pakistan	40.0% (800)	29.8% (596)	30.2% (603)
Portugal	41.3% (519)	21.0% (264)	37.7% (474)
Greece	41.7% (566)	24.6% (333)	33.7% (457)
Canada	41.9% (2,694)	29.9% (1,919)	28.2% (1,815)
Germany	42.1% (2,299)	23.8% (1,298)	34.1% (1,864)
Nigeria	43.0% (445)	23.5% (243)	33.5% (346)
Ireland	45.1% (615)	23.5% (321)	31.4% (429)
United Kingdom	48.0% (7,533)	25.6% (4,022)	26.4% (4,136)
Mexico	49.7% (645)	23.3% (302)	27.0% (351)
France	52.2% (2,875)	19.4% (1,067)	28.4% (1,563)
Taiwan	53.6% (684)	21.9% (280)	24.5% (312)
Singapore	54.0% (1,033)	17.8% (341)	28.1% (538)
Israel	55.3% (808)	19.2% (281)	25.5% (372)
Poland	56.1% (875)	15.6% (244)	28.3% (441)
Spain	59.4% (3,739)	15.8% (995)	24.8% (1,558)
South Korea	60.0% (1,175)	18.1% (354)	21.9% (429)
Japan	60.7% (1,696)	17.7% (494)	21.6% (604)
Italy	62.3% (8,411)	17.9% (2,423)	19.8% (2,676)
Brazil	63.1% (2,924)	18.2% (842)	18.8% (870)
United States	65.0% (29,160)	22.1% (9,896)	13.0% (5,823)
China	66.6% (10,978)	20.9% (3,442)	12.5% (2,065)
Iran	66.9% (2,773)	18.9% (784)	14.1% (585)
Indonesia	72.5% (903)	12.7% (158)	14.8% (185)
Turkey	72.7% (2,456)	11.0% (371)	16.3% (550)
India	72.8% (8,624)	14.6% (1,725)	12.6% (1,497)
