# Supplementary material for: How the scientific community responded to the COVID-19 pandemic: A subject-level time-trend bibliometric analysis
Source: PLoS One. 2021 Sep 30;16(9):e0258064. doi: 10.1371/journal.pone.0258064 (PMC8483337; doi:10.1371/journal.pone.0258064)
Supplement: S4 Fig — (PDF) [file pone.0258064.s016.pdf]

Supplementary Figure 4

		Open-Access
Region	Europe and Central Asia	75.6% (34,013)
	North America	73.2% (26,174)
	East Asia and Pacific	76.3% (19,357)
	South Asia	68.5% (7,543)
	Middle East and North Africa	71.7% (6,858)
	Latin America and Caribbean	77.6% (5,200)
	Sub-Saharan Africa	74.5% (2,721)
Income Group	High Income	74.3% (60,613)
	Upper-middle Income	75.1% (22,288)
	Lower-middle Income	69.7% (10,437)
	Low Income	83.0% (840)
International Collaboration	National	71.8% (65,462)
	Bi-national	76.0% (12,665)
	Multi-national	78.7% (7,227)
