# Supplementary material for: How the scientific community responded to the COVID-19 pandemic: A subject-level time-trend bibliometric analysis
Source: PLoS One. 2021 Sep 30;16(9):e0258064. doi: 10.1371/journal.pone.0258064 (PMC8483337; doi:10.1371/journal.pone.0258064)
Supplement: S5 Fig — (PDF) [file pone.0258064.s017.pdf]

Supplementary Figure 5

	Australia	Brazil	Canada	China	France	Germany	India	Iran	Italy	Japan	Netherlands	Pakistan	Saudi Arabia	South Africa	South Korea	Spain	Switzerland	Turkey	United Kingdom	United States
Annals Of The Rheumatic Diseases	4.40%	1.17%	2.93%	4.99%	16.13%	11.14%	2.64%	0.29%	24.63%	1.47%	7.04%			0.59%	0.88%	7.92%	3.23%	1.17%	9.09%	23.46%
BMJ (Clinical Research Ed.)	0.46%	0.23%	0.35%	0.35%	3.68%	0.12%	0.46%		0.12%		0.12%		0.12%	0.12%			0.12%		11.85%	2.76%
Clinical Infectious Diseases	2.66%	1.33%	2.66%	8.94%	5.32%	2.28%	1.14%		2.85%	1.90%	2.66%		0.38%	1.90%	0.57%	2.09%	2.47%	0.38%	6.08%	45.63%
Cureus		0.14%	0.71%	0.28%		0.28%	4.38%	0.28%	0.85%	0.42%		8.91%	2.26%	1.13%	0.14%	0.28%		0.28%	4.38%	60.54%
Dermatologic Therapy	3.65%	5.47%	1.30%	3.91%	0.52%	6.51%	19.53%	8.59%	33.59%			0.26%	0.78%	0.52%		5.47%	10.94%	17.97%	2.34%	22.66%
Disaster Medicine And Public Health Preparedness	1.14%	0.33%	1.46%	13.17%	1.46%	0.49%	6.83%	12.03%	6.99%	1.46%	1.14%	3.41%	2.11%	0.65%	1.79%	0.65%	0.98%	2.76%	4.72%	24.23%
Eclinicalmedicine	1.63%	2.17%	6.79%	16.85%	4.08%	3.26%	3.80%	0.27%	7.07%	0.54%	2.72%		0.27%	2.72%	0.54%	4.62%	1.90%	0.54%	20.38%	32.88%
Frontiers In Immunology	4.24%	6.50%	5.37%	16.67%	3.39%	9.60%	5.37%	1.13%	10.17%	1.13%	2.54%	0.56%	0.56%	0.56%	0.56%	5.93%	1.69%	0.56%	7.91%	28.81%
Frontiers In Medicine	2.35%	4.43%	2.49%	34.44%	4.98%	3.73%	2.35%	1.80%	11.48%	1.11%	1.24%	1.24%	2.07%	0.83%	1.38%	3.18%	1.66%	0.83%	6.36%	24.20%
Frontiers In Pharmacology	2.19%	0.82%	4.93%	30.96%	3.29%	1.92%	9.86%	3.84%	14.79%	1.37%	1.10%	1.64%	1.92%	3.56%	1.37%	5.48%	1.10%	0.82%	5.21%	19.73%
Frontiers In Psychiatry	3.94%	5.20%	5.02%	21.15%	1.43%	2.15%	5.91%	1.79%	20.97%	2.51%	3.76%	1.08%	1.43%	0.72%	1.43%	5.02%	2.51%	2.15%	14.52%	22.94%
Frontiers In Psychology	4.72%	3.18%	4.72%	15.09%	2.67%	5.24%	2.16%	0.41%	22.69%	0.31%	2.16%	2.67%	1.44%	1.03%	0.82%	13.35%	1.95%	2.36%	8.52%	20.74%
Frontiers In Public Health	8.41%	2.71%	4.34%	21.71%	3.12%	3.12%	7.73%	2.17%	11.26%	0.81%	0.54%	2.71%	3.80%	0.81%	2.04%	2.71%	2.99%	0.27%	10.85%	25.10%
Infection Control And Hospital Epidemiology	2.69%	2.89%	5.58%	10.33%	0.83%	1.45%	2.27%	3.31%	5.17%	1.86%	0.41%	1.45%	1.24%	1.03%	1.03%	1.86%	2.48%	0.21%	3.72%	51.24%
International Journal Of Environmental Research And Public Health	4.82%	2.44%	3.47%	14.97%	3.08%	5.91%	1.35%	1.09%	17.93%	3.86%	1.61%	1.61%	3.60%	1.54%	4.95%	12.08%	3.66%	0.58%	8.42%	17.22%
International Journal Of Infectious Diseases	2.47%	3.70%	1.94%	23.28%	5.64%	6.17%	4.41%	0.53%	11.64%	5.82%	2.29%	0.88%	2.82%	2.47%	4.59%	3.53%	2.82%	1.76%	9.70%	19.40%
JAMA	0.80%	0.80%	2.39%	3.59%	1.99%	1.79%	0.60%	0.20%	1.59%	0.60%	1.79%		0.20%	0.60%	0.40%	0.80%	2.19%		3.59%	66.14%
Journal Of Clinical Medicine	2.03%	1.52%	1.52%	1.77%	8.35%	7.85%	0.76%	0.76%	25.06%	6.84%	0.25%		0.51%	1.27%	4.30%	10.38%	3.54%	0.51%	3.80%	25.57%
Journal Of Infection	2.86%	1.67%	0.95%	37.23%	8.35%	2.39%	0.95%	0.48%	9.79%	3.58%	1.43%	1.43%	0.72%	0.24%	1.43%	5.25%	1.67%	0.48%	19.33%	6.21%
Journal Of Medical Internet Research	5.71%	1.14%	10.05%	25.57%	3.65%	6.16%	0.46%	0.68%	5.25%	0.91%	2.51%	1.37%	1.60%	0.23%	3.88%	4.11%	2.97%	0.23%	13.93%	36.30%
Journal Of Medical Virology	1.20%	3.49%	1.59%	30.68%	2.99%	1.99%	6.27%	2.99%	11.85%	2.99%	0.20%	3.19%	1.49%	0.50%	0.90%	2.19%	0.90%	4.48%	3.69%	19.62%
Medical Hypotheses	1.81%	4.54%	2.49%	3.40%	3.63%	2.49%	14.97%	5.22%	13.61%	0.91%	1.81%	1.13%	3.85%	0.91%	0.91%	1.81%	0.91%	5.67%	5.22%	18.59%
Nature	1.72%	0.98%	1.47%	6.63%	1.72%	4.42%	0.49%	0.25%	1.97%	0.74%	1.47%	0.25%	0.25%	1.47%	0.49%	0.49%	2.95%	0.25%	6.88%	15.48%
New England Journal Of Medicine	2.58%	1.88%	2.35%	5.40%	2.35%	3.99%	0.94%	0.23%	3.52%	1.41%	0.47%	0.23%	0.47%	1.64%	1.17%	1.88%	1.41%	0.23%	7.04%	59.62%
Plos One	3.38%	4.59%	5.39%	10.46%	4.67%	6.68%	3.30%	1.37%	7.08%	3.06%	2.49%	1.37%	1.85%	1.61%	1.93%	5.15%	3.54%	0.97%	10.62%	31.86%
Science Of The Total Environment	4.68%	5.15%	4.45%	25.29%	2.34%	3.28%	11.01%	2.58%	8.20%	5.15%	2.11%	1.64%	1.87%	0.47%	2.81%	8.43%	0.70%	1.41%	7.49%	23.89%
Scientific Reports	3.33%	3.33%	4.25%	15.53%	4.25%	6.84%	4.25%	1.85%	10.91%	5.73%	2.96%	0.55%	1.29%	0.55%	4.07%	4.44%	1.66%	0.74%	10.35%	28.47%
Sustainability (Switzerland)	3.51%	2.46%	3.51%	9.14%	1.58%	4.04%	1.58%	0.35%	13.71%	3.87%	1.23%	0.70%	2.46%	1.23%	7.03%	17.57%	0.35%	0.70%	4.39%	9.49%
The BMJ	3.35%	1.17%	2.33%	3.64%	0.44%	0.87%	1.75%		1.17%	0.44%	1.46%	0.58%	0.29%	1.60%	0.58%	0.73%	3.21%	0.29%	66.76%	13.56%
The Lancet	5.92%	4.08%	5.31%	11.84%	4.29%	10.00%	1.43%	0.20%	5.51%	1.02%	2.86%	0.61%	0.82%	5.31%	0.61%	4.49%	9.39%	0.41%	35.92%	32.24%
